# Discovery of 5-Phenoxy-2-aminopyridine Derivatives as Potent and Selective Irreversible Inhibitors of Bruton’s Tyrosine Kinase

**DOI:** 10.3390/ijms21218006

**Published:** 2020-10-28

**Authors:** Eun Lee, Hyewon Cho, Da Kyung Lee, JuHyun Ha, Byeong Jo Choi, Ji Hye Jeong, Jae-Ha Ryu, Jong Soon Kang, Raok Jeon

**Affiliations:** 1 College of Pharmacy, Sookmyung Women’s University, Seoul 04310, Korea; eunlee178@gmail.com (E.L.); hy-0802@sookmyung.ac.kr (H.C.); josephin914@sookmyung.ac.kr (D.K.L.); cu1006@sookmyung.ac.kr (J.H.); jjh4415@naver.com (J.H.J.); ryuha@sookmyung.ac.kr (J.-H.R.); 2 Laboratory Animal Resource Center, Korea Research Institute of Bioscience and Biotechnology, Ochang, Chungcheongbuk-do 28116, Korea; byung127@kribb.re.kr (B.J.C.); kanjon@kribb.re.kr (J.S.K.)

**Keywords:** Bruton’s tyrosine kinase, irreversible kinase inhibitor, hematological malignancy

## Abstract

As a member of the tyrosine protein kinase Tec (TEC) family, Bruton’s tyrosine kinase (BTK) is considered a promising therapeutic target due to its crucial roles in the B cell receptor (BCR) signaling pathway. Although many types of BTK inhibitors have been reported, there is an unmet need to achieve selective BTK inhibitors to reduce side effects. To obtain BTK selectivity and efficacy, we designed a novel series of type II BTK inhibitors which can occupy the allosteric pocket induced by the DFG-out conformation and introduced an electrophilic warhead for targeting Cys481. In this article, we have described the structure–activity relationships (SARs) leading to a novel series of potent and selective piperazine and tetrahydroisoquinoline linked 5-phenoxy-2-aminopyridine irreversible inhibitors of BTK. Compound **18g** showed good potency and selectivity, and its biological activity was evaluated in hematological tumor cell lines. The in vivo efficacy of **18g** was also tested in a Raji xenograft mouse model, and it significantly reduced tumor size, with 46.8% inhibition compared with vehicle. Therefore, we have presented the novel, potent, and selective irreversible inhibitor **18g** as a type II BTK inhibitor.

## 1. Introduction

Bruton’s tyrosine kinase (BTK), a member of the tyrosine protein kinase Tec (TEC) family, is expressed in B cells, myeloid cells, mast cells, and macrophages [1,2]. BTK is a crucial regulator of B cell development and function and is activated downstream of the B cell receptor (BCR) [3]. Abnormal functions of BTK are critical to the dysregulation of several steps in B cell proliferation, development, differentiation, and apoptosis, which induce overactive inflammation and hematologic malignancy [4].

**Figure 1:**
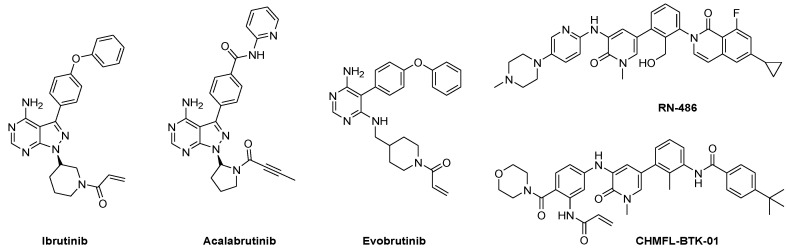
Examples of FDA-approved and preclinical BTK inhibitors.

Ibrutinib, the first US Food and Drug Administration (FDA)-approved BTK inhibitor (Figure 1), is an irreversible covalent inhibitor that targets Cys481, the residue adjacent to the ATP-binding site. It has been approved for the treatment of chronic lymphocytic leukemia (CLL), Waldenström macroglobulinemia, mantle cell lymphoma, and chronic graft versus host disease [5]. Another irreversible BTK inhibitor, acalabrutinib, was approved by the FDA for the treatment of mantle cell lymphoma, and several other covalent inhibitors are in clinical trials (Figure 1) [6]. Most of the adverse effects of ibrutinib are thought to be related to off-target effects arising from inhibition of TEC, janus kinase 3 (JAK3), and epidermal growth factor receptor kinase (EGFRK) not only the BTK [7]. Based on the location of the targeted Cys residue near the ATP-binding site, those kinases can be classified as Group 3F [8]. To overcome the issue of side effects, the discovery of highly selective BTK inhibitors is desirable.

Small molecule kinase inhibitors can be classified as reversible or irreversible inhibitors, depending on how they bind to the kinase [9]. Many BTK inhibitors, such as ibrutinib, acalabrutinib and evobrutinib, include a Michael acceptor with the aim of irreversible blocking by targeting the Cys residue at the BTK active site. Meanwhile, reversible inhibitors are generally further classified into four groups; type I to type IV inhibitors based on the conformation of the kinase ATP-binding pocket and where the inhibitor binds. Type I inhibitors bind to the kinase in adenosine triphosphate (ATP)-competitive mode at the ATP binding site. For type II inhibitors, the molecules interact with an inactive form of the target kinase and occupy both the ATP site and DFG pocket. Type III and type IV inhibitors are allosteric modulators which bind outside the ATP-binding site. RN-486, a representative reversible BTK inhibitor, can achieve BTK selectivity by targeting not only the adenine pockets but also a BTK specific allosteric pockets [10]. Recently, some hybridization strategies were attempted to convert reversible inhibitors to irreversible inhibitors, which resulted in selective inhibitors, such as CHMFL-BTK-01 [11].

Herein, we designed and synthesized aminopyridine derivatives as selective covalent BTK inhibitors and evaluated their antiproliferative activities in hematological tumor cell lines and their in vivo efficacy in a Raji xenograft mouse model. Consequently, we have identified a novel class of 5-phenoxy-2-aminopyridine compounds as potent and selective BTK inhibitors.

## 2. Results and Discussion

### 2.1. Drug Design

Ibrutinib contains a phenoxyphenyl moiety, which forms hydrophobic interactions with a selectivity pocket formed by Met449, Ile472, Leu542, and Phe540. The selectivity pocket is behind the Thr474 gatekeeper residue and is induced by the αC-helix-out conformation [12]. For this lipophilic substituent, acalabrutinib has a pyridylamide group instead of a phenoxy group. This particular structural change resulted in a greatly improved kinase selectivity profile [13], which showed that modifying the lipophilic group may be a good approach for targeting BTK selectively. Moreover, we hypothesized that expanding the lipophilic linker could induce not only the αC-helix-out conformation, but also the DFG-out conformation, which allows increased affinity and selectivity of compounds [14]. As there are currently no validated type II BTK inhibitors, the design of a type II inhibitor is a good starting point to achieve a novel and selective BTK inhibitor. We analyzed 74 BTK structures co-crystalized with small molecule inhibitors registered in the Protein Data Bank [15] (PDB IDs are listed in Supplementary Materials). Among the numerous activation states of BTK, there is a DFG-out conformation BTK structure complexed with a type II SRC kinase inhibitor (PDB ID: 3PJ3) [16], which provided the structural perspective for the design of type II BTK inhibitors.

**Figure 2:**
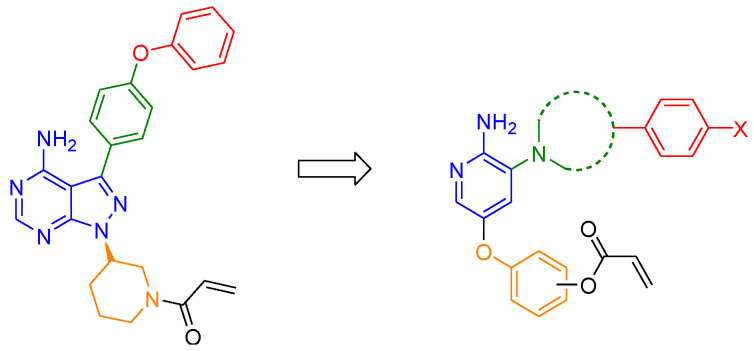
Design strategy for a novel series of aminopyridine derivatives as inhibitors of BTK. Red, lipophilic aryl substituent; green, lipophilic group linker; blue, hinge binder; orange, Michael acceptor linker.

When starting from ibrutinib, we considered it was necessary to revise the lipophilic group to target the allosteric site. To ensure flexibility for modification of the lipophilic group, the bicyclic pyrazolopyrimidine ring at the adenine binding pocket was replaced with a monocycle, such as evobrutinib (Figure 1). We also changed the lipophilic group linker phenyl ring of ibrutinib to a saturated piperazine ring to reduce structural rigidity (Figure 2). Furthermore, the monocyclic piperazine linker was also converted to a bicyclic tetrahydroisoquinoline linker to extend hydrophobic interactions deep into the allosteric pocket. In addition, the aromatic linker between the hinge binder and covalent warheads (Michael acceptor linker) can be positioned in the H2 pocket between Leu408 and Gly480 [17].

### 2.2. Synthesis

**Scheme 1:**
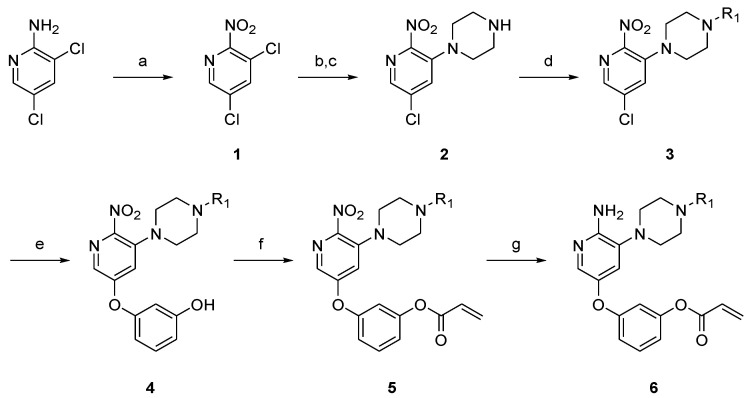
Synthesis of piperazine-linked aminopyridine derivatives. Reagents and conditions: (a) K_2_S_2_O_8_, H_2_SO_4_, 90 °C, 24 h, 45%; (b) 1-Boc-piperazine, K_2_CO_3_, toluene, 50 °C, 15 h, 42%; (c) trifluoroacetic acid (TFA), dichloromethane (DCM), rt, 4 h, 79%; (d) R_1_X, K_2_CO_3_, dimethylformamide (DMF), rt, 5 h, 40–93%; (e) resorcinol, Cs_2_CO_3_, DMF, 70 °C, 24 h, 20–79%; (f) acryloyl chloride, triethylamine (TEA), DCM, 0 °C, 3 h, 6–67%; (g) SnCl_2_∙2H_2_O, EtOH, 0 °C, 1 h, 4–23%.

Piperazine-linked aminopyridine derivatives were prepared as described in Scheme 1 and Supplementary Materials. From commercially available 2-amino-3,5-dichloropyridine, the amine group was converted to a nitro group using potassium persulfate, providing compound **1**. Nucleophilic substitution at the C3 position of pyridine **1** with *tert*-butyloxycarbonyl (Boc)-piperazine and deprotection of the Boc group with trifluoroacetic acid (TFA) afforded the 3-piperazinylpyridine **2**. *N*-Alkylation of the piperazine group with the appropriate aryl halide gave the corresponding nitropyridine **3**. Through a nucleophilic substitution reaction with cesium carbonate in dimethylformamide (DMF), resorcinol was introduced to the pyridine core scaffold and then acrylated with a Michael acceptor to achieve the intermediate **5**. Since the acryloyl group was removed under the initial reducing conditions that we tested, the nitro group was reduced to the amine using mild acidic conditions with tin (II) chloride, which afforded the piperazine-linked aminopyridine derivative **6**.

**Scheme 2:**
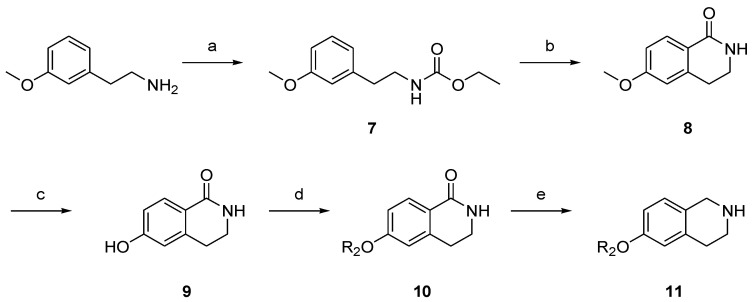
Synthesis of the tetrahydroisoquinoline scaffold for the lipophilic group linker. Reagents and conditions: (a) ethyl chloroformate, TEA, DCM, rt, 2 h, 100%; (b) polyphosphoric acid (PPA), 120 °C, 1.5 h, 68%; (c) 48% HBr, reflux, 24 h, 63%; (d) R_2_X, K_2_CO_3_, DMF, rt, 6 h, 27–55%; (e) LiAlH_4_, tetrahydrofuran (THF), reflux, 5 h, 40–95%.

**Scheme 3:**
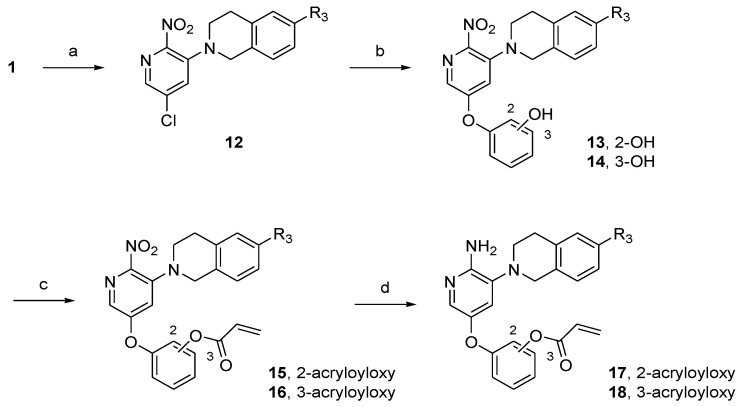
Synthesis of tetrahydroisoquinoline-linked aminopyridine derivatives. Reagents and conditions: (a) **11a–f** or 1,2,3,4-tetrahydroisoquinoline, K_2_CO_3_, toluene, 60 °C, 10 h, 34–57%; (b) resorcinol or catechol, K_2_CO_3_, dimethyl sulfoxide (DMSO), 18-crown-6, 100 °C, 3 h, 25–74%; (c) acryloyl chloride, TEA, DCM, rt, 1 h, 30–99%; (d) SnCl_2_∙2H_2_O, THF/EtOH, rt, 1 h, 4–67%.

To develop the bicyclic lipophilic group linker, tetrahydroisoquinoline scaffolds with aryl substituents were synthesized as depicted in Scheme 2. The commercially available 2-(3-methoxyphenyl)ethylamine was treated with ethyl chloroformate and triethylamine (TEA) in dichloromethane (DCM) to form carbamate **7**. The subsequent Bischler–Napieralski reaction with polyphosphoric acid (PPA) gave isoquinolinone **8**. Demethylation with HBr followed by nucleophilic substitution afforded intermediate **10**, which was then further reduced to tetrahydroisoquinoline with LiAlH_4_ to obtain lipophilic substituent **11**. The tetrahydroisoquinoline-linked aminopyridine derivatives **17** and **18** were synthesized in a similar manner to that of the piperazine derivatives (Scheme 3).

### 2.3. Structure–Activity Relationship Analysis

Synthesized aminopyridine derivatives were evaluated for in vitro BTK enzyme inhibitory activities. In vitro kinase assays were performed using commercially available ADP-Glo (Promega, Madison, WI, USA) and HotSpot Kinase assays (Reaction Biology Corporation, Malvern, PA, USA) at an ATP concentration of 10 μM. The SAR study of piperazine-linked aminopyridine **6** was performed (Table 1). Introduction of either the benzyl (**6b**–**d**) or phenylethyl (**6e**,**f**) group at the nitrogen of the piperazine led to better activity than the parent compound **6a**, suggesting the beneficial interactions of the substituted lipophilic groups in the lipophilic selectivity pocket. The chlorine substitution (**6d**) at the para position of the phenyl ring led to the highest potency within this series. However, the insertion of additional carbons (**6f**) between the piperazine and the terminal phenyl ring reduced the activity, implying a steric requirement for fitting into the selectivity pocket.

To identify a suitable linker moiety, the piperazine linker was replaced with tetrahydroisoquinoline. Eight of nine tetrahydroisoquinoline derivatives showed complete inhibition of enzyme activity in single dose determination (20 μM), which indicated improved potency compared with the piperazine derivatives, supporting our hypothesis (Table 2). In the case of tetrahydroisoquinoline derivatives, additional modifications resulted in direction toward the H2 pocket where the phenoxy linker-bearing Michael acceptor was positioned. The substitution position of the Michael acceptor significantly influenced the activity: the potency of the C2-substituted catechol-type compound **17a** was higher than that of C3-substituted resorcinol type **18a**; conversely, that of **17d** was less than that of **18d**.

In a series of resorcinol-type compounds, the effect of substituents at the C6 position of the tetrahydroisoquinoline ring was examined. The aromatic ring (**18d**) was favored, followed by *n*-propane (**18b**), cyclopropane (**18c**), or no substituent (**18a**). The benzyloxy-substituted compound **18d** was the most potent, with an IC_50_ value of 0.1 μM. Halogen substitution on the terminal phenyl ring (**18e** and **18f**) lowered potency (IC_50_ values of 8.5 μM and 1.0 μM, respectively). Meanwhile, methoxy substitution (**18g**) recovered the potency (IC_50_ = 0.2 μM).

Based on their sub-micromolar IC_50_ values, compounds **18d** and **18g** were selected for further study. The catechol-type compound **17d** was also chosen to compare the effects of the position of the acryloyloxy substitution. The kinase selectivity profiles for these compounds were explored over some TEC and SRC family kinases (Table 3). Overall, resorcinol-type compounds (**18d** and **18g**) showed better selectivity profiles than the catechol-type compound (**17d**) with lower kinase inhibition against EGFRK, interleukin-2-inducible T-cell kinase (ITK), TEC, and TXK tyrosine kinase (TXK). Compound **18g** was the most selective over EGFRK with 33.5% inhibition compared with **17d** and **18d** (87.0% and 60.5%, respectively). All three compounds significantly inhibited BMX/ETK and ERBB4. This off target activity could be a safety concern, but also provides the potential for additional applications.

### 2.4. Molecular Docking Analysis

**Figure 3:**
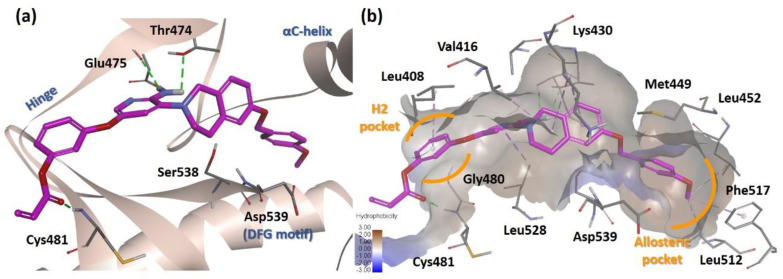
Docking analysis of **18g** (PDB ID: 3PJ3). (**a**) Predicted binding mode of **18g** to the kinase domain of BTK. Green dashed line, hydrogen bond interaction. (**b**) Hydrophobicity map of the active site. Purple dashed line, alkyl-alkyl interaction.

Molecular docking analysis of **18g** was performed based on the human BTK structure (PDB ID: 3PJ3), which has an allosteric pocket induced by the DFG-out conformation (Figure 3). The results suggested that the tetrahydroisoquinoline linker of **18g** placed the lipophilic moiety in the allosteric pocket of the BTK kinase domain as predicted for a type II inhibitor. The aminopyridine hinge binder forms two hydrogen bonds with the hinge residue Glu475 and the gatekeeper residue Thr474. The *para*-methoxybenzyl group is directed into the allosteric hydrophobic pocket formed by Met449, Leu452, Leu512, and Phe517. The orientation of the lipophilic substituent was stabilized by the teterahydroisoquinoline ring, which resulted in van der Waals interactions with Lys430. In addition, the Michael acceptor linker phenyl ring was located in the cleft between Leu408 and Gly480. This allowed hydrogen bonding between the carbonyl of the acryl group and the backbone of Cys481.

### 2.5. Effects on Experimental Models of Hematological Malignancy

Next, we examined the effects of the most potent compound **18g** and the representative catechol compound **17d** on two cell lines representative of hematological malignancy and compared them to ibrutinib. Both compounds **17d** and **18g** showed similar GI_50_ values in Ramos cell lines, and were less active than ibrutinib. In the Raji cell lines, **17d** exhibited a GI_50_ value lower than **18g** with an activity similar to ibrutinib (Table 4). All three compounds showed selective cytotoxicity in the two tested cancer cell lines over normal cells (MRC-5 and MCF10A). Among them, **18g** showed the most selective cytotoxicity against the tested cancer cell lines.

**Figure 4:**
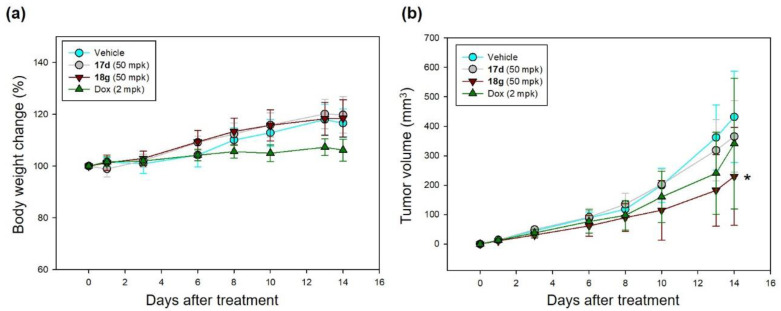
In vivo activity of **17d** and **18g** in Raji xenograft model. (**a**) Body weight change, (**b**) tumor size. Compounds were administered to Raji xenograft mice via i.p. (*n* = 5/group). Values are expressed as means ± standard deviation (SD): * *p* < 0.05 (vs. Vehicle); mpk, mg/kg.

We evaluated the in vivo efficacy of **17d** and **18g** in a Raji xenograft mouse model. Doxorubicin hydrochloride (Dox) was used as a positive control, and **17d** and **18g** were administered at a dose of 50 mg/kg once daily via i.p. injected for 2 weeks. There were no significant symptoms of toxicity based on clinical and necropsy findings. The control group showed slight weight loss, but not statistically significant, but no weight loss was observed in the **17d** and **18g** groups. Compound **18g** significantly reduced tumor size, with inhibition of 46.8% (229.5 ± 116.5 mm^3^) compared with vehicle (431.8 ± 155.2 mm^3^) (Figure 4).

## 3. Materials and Methods

### 3.1. Chemistry

Most reagents and solvents were purchased from commercial sources and used without further purification. Flash column chromatography was performed using silica gel 60 (Merck Millipore, Burlington, MA, USA, 230–400 mesh) with the indicated solvents. To monitor the completion of the reaction, thin-layer chromatography (TLC) was performed using Kieselgel 60 F254 plates (Merck Millipore, Burlington, MA, USA). IR spectra were recorded by a Nicolet iS5 spectrometer (Thermo Fisher Scientific, Waltham, MA, USA) using the ATR method. NMR spectra were recorded on an Avance III HD 500 spectrometer (Bruker, Billerica, MA, USA, ^1^H 500 MHz, and ^13^C 125 MHz). High performance liquid chromatography (HPLC) was used to evaluate compound purity. A Waters 1525 (Waters Corporation, Milford, MA, USA) system fitted with a reverse-phase column (Inertsil ODS-2 C18 column, 150 × 4.6 mm i.d., GS Sciences, Tokyo, Japan) was eluted with 75% methanol 1 ml/min and monitored at 254 nm. The purity was calculated based on the relative peak areas on the HPLC chromatogram. Mass spectra (ESI) were obtained using an Agilent single quadrupole LC-MS high-resolution mass spectrometer.

#### 3.1.1. General Procedures for the Synthesis of Compound **6**

To a mixture of compound **5** (1.0 equiv.) in EtOH at 0 °C, was added tin (II) chloride dihydrate (5.0 equiv.). After stirring at rt for 2 h, the mixture was basified with NaHCO_3_ (aq) and filtered onto a celite pad with MeOH. The combined organic layers were concentrated under reduced pressure and extracted with DCM. The combined organic layers were dried over MgSO_4_, filtered, and concentrated in vacuo. The residue was purified by flash column chromatography (*n*-hexane/EtOAc) to obtain compound **6**.

#### 3.1.2. Acrylic Acid 3-(6-Amino-5-piperazinylpyridin-3-yloxy)phenyl Ester (**6a**)

Brown solid (yield 4%); R*_f_* = 0.40 (EtOAc 100%); IR (neat, cm^−1^) 3340, 2923, 2852, 1735, 1640, 1595, 1466, 1367, 1335, 1272, 1244, 1176, 1137, 1043, 993, 967; ^1^H NMR (500 MHz, CDCl_3_) δ 7.63 (1H, br s), 7.14 (1H, t, *J* = 8.0 Hz), 6.96 (1H, d, *J* = 2.5 Hz), 6.58 (1H, dd, *J* = 10.5, 17.0 Hz), 6.55 (1H, dd, *J* = 2.0, 8.0 Hz), 6.49 (1H, dd, *J* = 2.0, 8.0 Hz), 6.38 (1H, t, *J* = 2.0 Hz), 6.33 (1H, dd, *J* = 1.5, 17.0 Hz), 5.75 (1H, dd, *J* = 1.5, 10.5 Hz), 4.90 (2H, br s), 3.81 (2H, br s), 3.71 (2H, br s), 2.90 (4H, br s); ^13^C NMR (125 MHz, CDCl_3_) δ 165.8, 159.7, 157.8, 151.0, 150.2, 134.5, 133.7, 130.7, 128.9, 127.2, 121.1, 110.3, 109.2, 104.4, 51.0, 50.5, 46.3, 42.5; LCMS (ESI) *m*/*z* calcd for C_18_H_20_N_4_O_3_, 340.15; Found 341.5 [M + H]^+^.

#### 3.1.3. Acrylic Acid 3-[6-Amino-5-(4-benzylpiperazinyl)pyridin-3-yloxy]phenyl Ester (**6b**)

Brown solid (yield 15%); R*_f_* = 0.44 (*n-*hexane/EtOAc = 1:5); IR (neat, cm^−1^) 3058, 2942, 2820, 1664, 1594, 1462, 1369, 1336, 1265, 1214, 1178, 1134, 996, 737, 700; ^1^H NMR (500 MHz, CDCl_3_) δ 7.69 (1H, d, *J* = 2.5 Hz), 7.34–7.26 (6H, m), 6.98 (1H, d, *J* = 2.5 Hz), 6.83–6.80 (2H, m), 6.70 (1H, t, *J* = 2.0 Hz), 6.58 (1H, dd, *J* = 1.0, 17.5 Hz), 6.28 (1H, dd, *J* = 10.5, 17.5 Hz), 6.00 (1H, dd, *J* = 1.0, 10.5 Hz), 4.63 (2H, br s), 3.58 (2H, s), 2.93 (4H, br s), 2.60 (4H, br s); ^13^C NMR (125 MHz, CDCl_3_) δ 164.3, 159.7, 151.6, 151.4, 145.1, 138.0, 135.2, 134.3, 132.8, 130.1, 129.3, 128.4, 127.9, 127.3, 120.1, 115.6, 114.2, 110.4, 63.1, 53.5, 50.6; LCMS (ESI) *m*/*z* calcd for C_25_H_26_N_4_O_3_, 430.20; Found 431.7 [M + H]^+^.

#### 3.1.4. Acrylic Acid 3-{6-Amino-5-[4-(4-fluorobenzyl)piperazinyl]pyridin-3-yloxy}phenyl Ester (**6c**)

Brown solid (yield 12%); R*_f_* = 0.32 (*n-*hexane/EtOAc = 1.5:1); IR (neat, cm^−1^) 2939, 2821, 1740, 1602, 1507, 1479, 1402, 1366, 1312, 1243, 1220, 1157, 1025, 1002, 841, 796, 736, 700; ^1^H NMR (500 MHz, CDCl_3_) δ 7.68 (1H, d, *J* = 2.5 Hz), 7.31–7.27 (3H, m), 7.01 (2H, t, *J* = 8.5 Hz), 6.98 (1H, d, *J* = 2.5 Hz), 6.81 (2H, t, *J* = 8.5 Hz), 6.70 (1H, t, *J* = 2.5 Hz), 6.58 (1H, dd, *J* = 1.0, 17.5 Hz), 6.28 (1H, dd, *J* = 10.5, 17.5 Hz), 6.00 (1H, dd, *J* = 1.0, 10.5 Hz), 4.71 (2H, br s), 3.53 (2H, s), 2.93 (4H, br s), 2.58 (4H, br s); ^13^C NMR (125 MHz, CDCl_3_) δ 164.3, 162.2 (d, *J_C–F_* = 243.9 Hz), 159.7, 151.7, 151.5, 145.1, 135.3, 134.2, 133.7 (d, *J_C–F_* = 3.1 Hz), 132.9, 130.7 (d, *J_C–F_* = 7.9 Hz), 130.2, 127.9, 120.3, 115.6, 115.2 (d, *J_C–F_* = 21.1 Hz), 114.2, 110.4, 62.3, 53.5, 50.6; LCMS (ESI) *m*/*z* calcd for C_25_H_25_FN_4_O_3_, 448.19; Found 449.6 [M + H]^+^.

#### 3.1.5. Acrylic Acid 3-{6-Amino-5-[4-(4-chlorobenzyl)piperazinyl]pyridin-3-yloxy}phenyl Ester (**6d**)

Brown solid (yield 10%); R*_f_* = 0.32 (*n-*hexane/EtOAc = 1.5:1); IR (neat, cm^−1^) 3048, 2940, 2821, 1742, 1594, 1488, 1402, 1367, 1311, 1293, 1247, 1212, 1157, 1086, 1002, 795, 736; ^1^H NMR (500 MHz, CDCl_3_) δ 7.69 (1H, d, *J* = 2.5 Hz), 7.32–7.26 (5H, m), 6.98 (1H, d, *J* = 2.5 Hz), 6.83–6.80 (2H, m), 6.70 (1H, t, *J* = 2.5 Hz), 6.58 (1H, dd, *J* = 1.0, 17.5 Hz), 6.28 (1H, dd, *J* = 10.5, 17.5 Hz), 6.00 (1H, dd, *J* = 1.0, 10.5 Hz), 4.63 (2H, br s), 3.53 (2H, s), 2.93 (4H, br s), 2.58 (4H, br s); ^13^C NMR (125 MHz, CDCl_3_) δ 164.4, 159.8, 151.7, 151.5, 145.1, 136.6, 135.2, 134.5, 133.0, 132.9, 130.5, 130.2, 128.6, 127.9, 120.2, 115.6, 114.2, 110.4, 62.3, 53.5, 50.6; LCMS (ESI) *m*/*z* calcd for C_25_H_25_ClN_4_O_3_, 464.16; Found 465.6 [M + H]^+^.

#### 3.1.6. Acrylic Acid 3-[6-Amino-5-(4-phenethylpiperazinyl)pyridin-3-yloxy]phenyl Ester (**6e**)

Brown solid (yield 19%); R*_f_* = 0.48 (EtOAc 100%); IR (neat, cm^−1^) 3025, 2942, 2819, 1743, 1608, 1464, 1402, 1372, 1336, 1247, 1152, 1023, 1001, 775, 735, 700; ^1^H NMR (500 MHz, CDCl_3_) δ 7.70 (1H, d, *J* = 2.5 Hz), 7.31–7.28 (3H, m), 7.23–7.21 (3H, m), 7.00 (1H, d, *J* = 2.5 Hz), 6.83–6.81 (2H, m), 6.71 (1H, t, *J* = 2.5 Hz), 6.59 (1H, dd, *J* = 1.0, 17.5 Hz), 6.29 (1H, dd, *J* = 10.5, 17.5 Hz), 6.01 (1H, dd, *J* = 1.0, 10.5 Hz), 4.65 (2H, br s), 2.97 (4H, br s), 2.85–2.82 (2H, m), 2.69–2.66 (6H, m); ^13^C NMR (125 MHz, CDCl_3_) δ 164.4, 159.7, 151.7, 151.4, 145.1, 140.3, 135.2, 134.4, 132.9, 130.2, 128.8, 128.6, 127.9, 126.3, 120.2, 115.6, 114.2, 110.4, 60.6, 53.7, 50.6, 33.8; LCMS (ESI) *m*/*z* calcd for C_26_H_28_N_4_O_3_, 444.22; Found 445.6 [M + H]^+^.

#### 3.1.7. Acrylic Acid 3-(6-Amino-5-{4-[2-(4-chlorophenyl)ethyl]piperazinyl}pyridin-3-yloxy)phenyl Ester (**6f**)

Brown solid (yield 4%); R*_f_* = 0.24 (*n-*hexane/EtOAc = 1:50); IR (neat, cm^−1^) 3355, 2938, 2821, 1742, 1608, 1464, 1402, 1336, 1247, 1154, 1002, 776; ^1^H NMR (500 MHz, CDCl_3_) δ 7.69 (1H, d, *J* = 2.5 Hz), 7.29 (1H, t, *J* = 8.5 Hz), 7.25 (2H, d, *J* = 8.5 Hz), 7.14 (2H, d, *J* = 8.5 Hz), 6.99 (2H, d, *J* = 2.5 Hz), 6.83–6.80 (2H, m), 6.71 (1H, t, *J* = 2.5 Hz), 6.58 (1H, dd, *J* = 1.0, 17.5 Hz), 6.29 (1H, dd, *J* = 10.5, 17.5 Hz), 6.01 (1H, dd, *J* = 1.0, 10.5 Hz), 4.68 (2H, br s), 2.96 (4H, br s), 2.81–2.78 (2H, m), 2.65–2.62 (6H, m); LCMS (ESI) *m/z* calcd for C_26_H_27_ClN_4_, 478.18; Found 479.7 [M + H]^+^.

#### 3.1.8. 4-[5-(3-Acryloyloxyphenoxy)-2-aminopyridin-3-yl]piperazine carboxylic Acid Benzyl Ester (**6g**)

Brown solid (yield 23%); R*_f_* = 0.36 (*n-*hexane/EtOAc = 1:5); IR (neat, cm^−1^) 3351, 3062, 2827, 1742, 1688, 1608, 1466, 1403, 1361, 1333, 1284, 1245, 1152, 1087, 1044, 992, 908, 735, 699; ^1^H NMR (500 MHz, CDCl_3_) δ 7.72 (1H, d, *J* = 2.5 Hz), 7.39–7.32 (5H, m), 7.29 (1H, t, *J* = 8.0 Hz), 6.95 (1H, d, *J* = 2.5 Hz), 6.82 (2H, td, *J* = 2.0, 8.0 Hz), 6.70 (1H, t, *J* = 2.5 Hz), 6.58 (1H, dd, *J* = 1.0, 17.5 Hz), 6.28 (1H, dd, *J* = 10.5, 17.5 Hz), 6.01 (1H, dd, *J* = 1.0, 10.5 Hz), 5.16 (2H, s), 4.65 (2H, br s), 3.65 (4H, br s), 2.88 (4H, br s); ^13^C NMR (125 MHz, CDCl_3_) δ 164.3, 159.6, 155.4, 151.7, 151.3, 145.2, 136.7, 135.1, 134.6, 133.0, 130.3, 128.7, 128.3, 128.1, 127.9, 120.4, 115.7, 114.3, 110.5, 67.5, 50.5, 44.4; LCMS (ESI) *m*/*z* calcd for C_26_H_26_N_4_O_5_, 474.19; Found 475.7 [M + H]^+^.

#### 3.1.9. General Procedures for the Synthesis of Compounds **17** and **18**

To a mixture of compound **15** or **16** (1.0 equiv.) in THF/EtOH (2:1) was added tin (II) chloride dihydrate (5.0 equiv.). After stirring at rt for 2 h, the mixture was basified with NaHCO_3_ (aq) and filtered onto a celite pad with MeOH. The combined organic layers were concentrated under reduced pressure and extracted with EtOAc. The combined organic layers were dried over MgSO_4_, filtered, and concentrated in vacuo. The residue was purified by flash column chromatography (*n*-hexane/EtOAc) to obtain compound **17** or **18**.

#### 3.1.10. Acrylic Acid 2-[6-Amino-5-(3,4-dihydro-1*H*-isoquinolin-2-yl)pyridin-3-yloxy]phenyl Ester (**17a**)

Yellow solid (yield 31%); R*_f_* = 0.60 (*n-*hexane/EtOAc = 1:1); IR (neat, cm^−1^) 2980, 2888, 1596, 1460, 1380, 1315, 1230, 1315, 1238, 1150, 1023, 971; ^1^H NMR (500 MHz, CDCl_3_) δ 7.69 (1H, d, *J* = 2.5 Hz), 7.21–7.14 (5H, m), 7.10–7.06 (2H, m), 7.03 (1H, d, *J* = 2.5 Hz), 6.89 (1H, dd, *J* = 1.5, 8.5 Hz), 6.59 (1H, dd, *J* = 1.0, 17.5 Hz), 6.32 (1H, dd, *J* = 10.5, 17.5 Hz), 6.00 (1H, dd, *J* = 1.0, 10.5 Hz), 4.66 (2H, br s), 4.06 (2H, s), 3.22 (2H, t, *J* = 6.0 Hz), 3.01 (2H, t, *J* = 6.0 Hz); ^13^C NMR (125 MHz, CDCl_3_) δ 164.1, 151.4, 150.2, 145.8, 140.8, 135.1, 134.5, 133.9, 132.9, 129.1, 127.6, 127.1, 126.6, 123.7, 123.3, 119.9, 118.0, 53.2, 48.9, 29.8; LCMS (ESI) *m*/*z* calcd for C_23_H_21_N_3_O_3_, 387.16; Found 388.16 [M + H]^+^.

#### 3.1.11. Acrylic Acid 2-[6-Amino-5-(6-benzyloxy-3,4-dihydro-1*H*-isoquinolin-2-yl)pyridin-3-yloxy]phenyl Ester (**17d**)

Ivory solid (yield 23%); R*_f_* = 0.52 (*n-*hexane/EtOAc = 1:1); IR (neat, cm^−1^) 2980, 2888, 1735, 1610, 1461, 1380, 1239, 1150, 1015, 969; ^1^H NMR (500 MHz, CDCl_3_) δ 7.68 (1H, d, *J* = 2.5 Hz), 7.42 (2H, d, *J* = 7.5 Hz), 7.38 (2H, t, *J* = 7.5 Hz), 7.31 (1H, t, *J* = 7.5 Hz), 7.15 (2H, t, *J* = 7.5 Hz), 7.07 (1H, t, *J* = 7.5 Hz), 7.01 (1H, d, *J* = 2.5 Hz), 6.97 (1H, d, *J* = 8.5 Hz), 6.88 (1H, d, *J* = 8.0 Hz), 6.81–6.77 (2H, m), 6.57 (1H, dd, *J* = 1.0, 17.0 Hz), 6.31 (1H, dd, *J* = 10.5, 17.0 Hz), 5.98 (1H, dd, *J* = 1.0, 10.5 Hz), 5.04 (2H, s), 4.72 (2H, br s), 3.98 (2H, s), 3.17 (2H, t, *J* = 5.5 Hz), 2.95 (2H, t, *J* = 5.5 Hz); ^13^C NMR (125 MHz, CDCl_3_) δ 164.1, 159.8, 157.6, 154.5, 151.4, 150.2, 145.7, 140.7, 137.2, 135.1, 135.1, 133.7, 132.9, 130.2, 128.7, 128.1, 127.5, 127.5, 127.1, 126.9, 123.6, 123.2, 119.8, 117.9, 114.7, 113.2, 70.2, 52.7, 48.7, 30.0; LCMS (ESI) *m*/*z* calcd for C_30_H_27_N_3_O_4_, 493.20; Found 494.20 [M + H]^+^; purity 97%.

#### 3.1.12. Acrylic Acid 3-[6-Amino-5-(3,4-dihydro-1*H*-isoquinolin-2-yl)pyridin-3-yloxy]phenyl Ester (**18a**)

Yellow solid (yield 30%); R*_f_* = 0.60 (*n-*hexane/EtOAc = 1:1); IR (neat, cm^−1^) 2980, 2888, 1461, 1382, 1239, 1151, 966; ^1^H NMR (500 MHz, CDCl_3_) δ 7.73 (1H, d, *J* = 2.5 Hz), 7.30 (1H, t, *J* = 8.0 Hz), 7.21–7.15 (3H, m), 7.07 (1H, d, *J* = 7.0 Hz), 7.06 (1H, d, *J* = 2.5 Hz), 6.83 (1H, dd, *J* = 2.5, 8.5 Hz), 6.82 (1H, dd, *J* = 2.0, 8.5 Hz), 6.73 (1H, dd, *J* = 2.0, 2.5 Hz), 6.59 (1H, d, *J* = 1.5, 17.5 Hz), 6.29 (1H, dd, *J* = 10.5, 17.5 Hz), 6.01 (1H, d, *J* = 1.5, 10.5 Hz), 4.73 (2H, br s), 4.09 (2H, s), 3.24 (2H, t, *J* = 6.0 Hz), 3.02 (2H, t, *J* = 6.0 Hz); ^13^C NMR (125 MHz, CDCl_3_) δ 164.4, 159.7, 151.8, 151.6, 144.9, 135.4, 134.3, 133.8, 133.8, 132.9, 130.2, 129.1, 127.9, 126.7, 126.5, 126.1, 120.9, 115.7, 114.2, 110.4, 53.3, 48.9, 29.6; LCMS (ESI) *m*/*z* calcd for C_23_H_21_N_3_O_3_, 387.16; Found 388.16 [M + H]+.

#### 3.1.13. Acrylic Acid 3-[6-Amino-5-(6-propoxy-3,4-dihydro-1*H*-isoquinolin-2-yl)pyridin-3-yloxy]phenyl Ester (**18b**)

Yellow solid (yield 23%); R*_f_* = 0.56 (*n-*hexane/EtOAc = 1:1); IR (neat, cm^−1^) 2980, 2883, 1595, 1459, 1399, 1381, 1314, 1237, 1151, 1023, 981; ^1^H NMR (500 MHz, CDCl_3_) δ 7.72 (1H, d, *J* = 2.5 Hz), 7.30 (1H, t, *J* = 8.5 Hz), 7.05 (1H, d, *J* = 2.5 Hz), 6.98 (1H, d, *J* = 8.5 Hz), 6.83–6.81 (2H, m), 6.75–6.70 (3H, m), 6.58 (1H, dd, *J* = 1.0, 17.5 Hz), 6.29 (1H, dd, *J* = 10.5, 17.5 Hz), 6.00 (1H, dd, *J* = 1.0, 10.5 Hz), 4.73 (2H, br s), 4.02 (2H, s), 3.91 (2H, t, *J* = 7.0 Hz), 3.21 (2H, t, *J* = 6.0 Hz), 2.97 (2H, t, *J* = 6.0 Hz), 1.80 (2H, sext., *J* = 7.0 Hz), 1.03 (3H, t, *J* = 7.0 Hz); ^13^C NMR (125 MHz, CDCl_3_) δ 164.3, 159.6, 157.8, 151.6, 151.5, 144.9, 135.3, 134.9, 133.8, 133.2, 132.8, 130.2, 127.8, 127.3, 126.2, 125.4, 120.9, 115.5, 114.3, 114.1, 110.2, 52.7, 52.0, 51.5, 48.7, 29.2; LCMS (ESI) *m*/*z* calcd for C_26_H_27_N_3_O_4_, 445.20; Found 446.21 [M + H]^+^.

#### 3.1.14. Acrylic Acid 3-[6-Amino-5-(6-cyclopropylmethoxy-3,4-dihydro-1*H*-isoquinolin-2-yl)pyridin-3-yloxy]phenyl Ester (**18c**)

Yellow solid (yield 67%); R*_f_* = 0.60 (*n-*hexane/EtOAc = 1:1); IR (neat, cm^−1^) 2981, 2884, 1595, 1459, 1399, 1369, 1311, 1276, 1237, 1153, 1129, 1021; ^1^H NMR (500 MHz, CDCl_3_) δ 7.70 (1H, d, *J* = 2.5 Hz), 7.30 (1H, t, *J* = 8.0 Hz), 7.05 (1H, d, *J* = 2.5 Hz), 6.98 (1H, d, *J* = 8.5 Hz), 6.82 (2H, dd, *J* = 2.5, 8.5 Hz), 6.76–6.70 (3H, m), 6.59 (1H, dd, *J* = 1.5, 17.5 Hz), 6.29 (1H, dd, *J* = 10.5, 17.5 Hz), 6.01 (1H, dd, *J* = 1.5, 10.5 Hz), 4.79 (2H, br s), 4.02 (2H, s), 3.79 (2H, d, *J* = 7.0 Hz), 3.21 (2H, t, *J* = 6.0 Hz), 2.97 (2H, t, *J* = 6.0 Hz), 1.30–1.23 (1H, m), 0.64 (2H, dd, *J* = 5.0, 12.5 Hz), 0.34 (2H, dd, *J* = 5.0, 10.0 Hz); ^13^C NMR (125 MHz, CDCl_3_) δ 164.3, 159.6, 157.7, 151.6, 151.5, 144.9, 135.3, 134.9, 133.9, 132.8, 130.1, 127.8, 127.7, 127.4, 126.4, 120.9, 120.6, 115.5, 114.3, 114.1, 113.0, 110.3, 52.7, 50.7, 48.6, 40.6, 29.8; LCMS (ESI) *m*/*z* calcd for C_27_H_27_N_3_O_4_, 457.20; Found 458.22 [M + H]^+^.

#### 3.1.15. Acrylic Acid 3-[6-Amino-5-(6-benzyloxy-3,4-dihydro-1*H*-isoquinolin-2-yl)pyridin-3-yloxy]phenyl Ester (**18d**)

Yellow solid (yield 63%); R*_f_* = 0.61 (*n-*hexane/EtOAc = 1:1); IR (neat, cm^−1^) 2980, 1739, 1607, 1462, 1401, 1378, 1313, 1237, 1151, 1023, 1001; ^1^H NMR (500 MHz, CDCl_3_) δ 7.72 (1H, d, *J* = 2.5 Hz), 7.43 (2H, d, *J* = 7.0 Hz), 7.38 (2H, t, *J* = 7.5 Hz), 7.33–7.28 (2H, m), 7.04 (1H, d, *J* = 2.5 Hz), 6.99 (1H, d, *J* = 8.0 Hz), 6.83–6.78 (4H, m), 6.72 (1H, t, *J* = 2.5 Hz), 6.58 (1H, dd, *J* = 1.0, 17.5 Hz), 6.29 (1H, dd, *J* = 10.5, 17.5 Hz), 6.00 (1H, dd, *J* = 1.5, 10.5 Hz), 5.05 (2H, s), 4.72 (2H, br s), 4.02 (2H, s), 3.21 (2H, t, *J* = 6.0 Hz), 2.97 (2H, t, *J* = 6.0 Hz); ^13^C NMR (125 MHz, CDCl_3_) δ 164.4, 159.8, 157.6, 154.5, 151.7, 151.6, 145.0, 137.2, 135.2, 135.1, 134.5, 132.9, 130.2, 128.7, 128.1, 127.9, 127.6, 127.5, 126.9, 120.5, 115.6, 114.8, 114.2, 113.3, 110.4, 70.2, 52.8, 48.7, 29.9; LCMS (ESI) *m*/*z* calcd for C_30_H_27_N_3_O_4_, 493.20; Found 494.21 [M + H]^+^.

#### 3.1.16. Acrylic Acid 3-{6-Amino-5-[6-(4-fluorobenzyloxy)-3,4-dihydro-1*H*-isoquinolin-2-yl]pyridin-3-yloxy}phenyl Ester (**18e**)

Yellow solid (yield 4%); R*_f_* = 0.60 (*n-*hexane/EtOAc = 1:1); IR (neat, cm^−1^) 3321, 2922, 2850, 1735, 1603, 1508, 1460, 1400, 1379, 1235, 1151, 1022; ^1^H NMR (500 MHz, CDCl_3_) δ 7.71 (1H, d, *J* = 2.0 Hz), 7.42 (2H, m), 7.32 (1H, t, *J* = 8.0 Hz), 7.09 (3H, m), 7.00 (1H, d, *J* = 8.5 Hz), 6.84 (2H, m), 6.81 (1H, dd, *J* = 2.0, 8.5 Hz), 6.77 (1H, d, *J* = 2.0 Hz), 6.73 (1H, d, *J* = 2.0 Hz), 6.57 (1H, d, *J* = 17.0 Hz), 6.32 (1H, dd, *J* = 10.5, 17.0 Hz), 6.02 (1H, d, *J* = 10.5 Hz), 5.01 (2H, s), 4.82 (2H, br s), 4.03 (2H, s), 3.23 (2H, t, *J* = 6.0 Hz), 2.99 (2H, t, *J* = 6.0 Hz); ^13^C NMR (125 MHz, CDCl_3_) δ 164.4, 159.7, 157.4, 151.8, 151.7, 145.0, 135.4, 132.9, 130.2, 129.4, 129.4, 127.9, 127.6, 127.0, 120.8, 115.9, 115.7, 115.7, 115.5, 115.3, 114.8, 114.2, 113.3, 110.4, 69.5, 53.6, 52.8, 48.7, 29.9; LCMS (ESI) *m*/*z* calcd for C_30_H_25_FN_3_O_4_, 511.19; Found 512.19 [M + H]^+^.

#### 3.1.17. Acrylic Acid 3-{6-Amino-5-[6-(4-chlorobenzyloxy)-3,4-dihydro-1*H*-isoquinolin-2-yl]pyridin-3-yloxy}phenyl Ester (**18f**)

Yellow solid (yield 55%); R*_f_* = 0.62 (*n-*hexane/EtOAc = 1:1); IR (neat, cm^−1^) 2980, 2952, 2888, 1458, 1443, 1234, 1148, 1015; ^1^H NMR (500 MHz, CDCl_3_) δ 7.72 (1H, d, *J* = 2.5 Hz), 7.36 (4H, s), 7.30 (1H, t, *J* = 8.5 Hz), 7.05 (1H, d, *J* = 2.5 Hz), 7.00 (1H, d, *J* = 8.5 Hz), 6.83 (1H, d, *J* = 8.0 Hz), 6.82 (1H, d, *J* = 8.0 Hz), 6.79 (1H, dd, *J* = 2.5, 8.0 Hz), 6.76 (1H, d, *J* = 2.5 Hz), 6.72 (1H, t, *J* = 2.5 Hz), 6.59 (1H, dd, *J* = 1.0, 17.0 Hz), 6.29 (1H, dd, *J* = 10.0, 17.0 Hz), 6.01 (1H, dd, *J* = 1.0, 10.0 Hz), 5.02 (2H, s), 4.72 (2H, br s), 4.03 (2H, s), 3.22 (2H, t, *J* = 5.5 Hz), 2.98 (2H, t, *J* = 5.5 Hz); ^13^C NMR (125 MHz, CDCl_3_) δ 164.4, 159.8, 157.3, 151.7, 151.6, 145.0, 135.7, 135.2, 134.4, 133.9, 132.9, 130.2, 128.9, 128.8, 128.7, 127.9, 127.6, 126.9, 120.6, 115.6, 114.8, 114.2, 113.2, 110.4, 69.4, 52.8, 48.7, 29.9; LCMS (ESI) *m*/*z* calcd for C_30_H_25_ClN_3_O_4_, 527.16; Found 528.16 [M + H]^+^.

#### 3.1.18. Acrylic Acid 3-{6-Amino-5-[6-(4-methoxybenzyloxy)-3,4-dihydro-1*H*-isoquinolin-2-yl]pyridin-3-yloxy}phenyl Ester (**18g**)

Yellow solid (yield 42%); R*_f_* = 0.57 (*n-*hexane/EtOAc = 1:1); IR (neat, cm^−1^) 3345, 2980, 2970, 2930, 1740, 1609, 1513, 1462, 1400, 1379, 1304, 1241, 1153, 1025, 1001; ^1^H NMR (500 MHz, CDCl_3_) δ 7.70 (1H, d, *J* = 2.5 Hz), 7.33 (2H, d, *J* = 8.5 Hz), 7.28 (1H, t, *J* = 8.0 Hz), 7.03 (1H, d, *J* = 2.5 Hz), 6.96 (1H, d, *J* = 8.5 Hz), 6.90 (2H, d, *J* = 8.5 Hz), 6.82–6.77 (3H, m), 6.76 (1H, d, *J* = 2.0 Hz), 6.73 (1H, t, *J* = 2.5 Hz), 6.57 (1H, dd, *J* = 1.0, 17.5 Hz), 6.27 (1H, dd, *J* = 10.5, 17.5 Hz), 5.98 (1H, dd, *J* = 1.0, 10.5 Hz), 4.95 (2H, s), 4.82 (2H, br s), 4.00 (2H, s), 3.78 (3H, s), 3.18 (2H, t, *J* = 6.0 Hz), 2.95 (2H, t, *J* = 6.0 Hz); ^13^C NMR (125 MHz, CDCl_3_) δ 164.4, 159.8, 159.6, 157.7, 151.7, 151.7, 145.0, 135.3, 135.1, 134.4, 132.9, 130.2, 129.3, 129.2, 127.9, 127.5, 126.8, 120.6, 115.6, 114.8, 114.2, 114.2, 113.3, 110.4, 70.0, 55.5, 52.8, 48.8, 29.9; LCMS (ESI) *m*/*z* calcd for C_31_H_29_N_3_O_5_, 523.21; Found 524.22 [M + H]^+^; purity 95%.

### 3.2. BTK Kinase Assay

#### 3.2.1. ADP-Glo Assay

To examine the enzyme activity, a kinase assay was performed using the ADP-Glo kinase assay kit (Promega, Madison, WI, USA) and the BTK enzyme system (Promega, Madison, WI, USA). Kinase reactions were performed using 10 μM ATP. The amount of the enzyme was determined as the concentration giving a signal-to-background ratio of 10 by titrating before using each enzyme batch. For 30 min, the inhibitor and enzyme in the kinase buffer were pre-incubated at room temperature. The ATP/substrate mixture was added and incubated for 30 min in a total kinase reaction volume of 10 μL at room temperature. The kinase reaction solution and ADP-Glo reagent were mixed in a 1:1 ratio (total 20 μL). After incubation for 30 min at room temperature, a kinase detection reagent (20 μL) was added for another 30 min. The luminescence intensity was read on a GloMax® Discover (Promega, Madison, WI, USA). For the single-dose screening enzyme inhibition assays, compounds were tested at 20 μM. Since the staurosporine completely inhibits BTK at 20 μM, it was used as the positive control and the relative percentage inhibition value was calculated compared to staurosporine as 100%. The IC_50_ values were measured for a 7-dose, 10-fold serial dilution starting at 100 μM, and then calculated using four parameter logistic standard curve fitting using Sigmaplot software ver. 12.0.

#### 3.2.2. HotSpot Kinase Assay

In vitro profiling of BTK, BMX, EGFRK, ERBB2, ERBB4, ITK, JAK3, TEC, and TXK kinases was performed at Reaction Biology Corporation (Malvern, PA, USA) [18]. The kinase and substrate pairs were prepared in reaction buffer: 20 mM HEPES at pH 7.5, 10 mM MgCl_2_, 1 mM EGTA, 0.02% Brij 35, 0.02 mg/mL BSA, 0.1 mM Na_3_VO_4_, 2 mM DTT, and 1% DMSO. Compounds were added into the kinase reaction mixture and incubated for 20 min. Then, ^33^P-ATP was delivered to the reaction mixture to initiate the reaction which was carried out at 10 μM total ATP. After 2 h at room temperature, the reaction mixture was spotted onto P81 ion exchange paper, and then kinase activity was detected by the filter-binding method. Compounds were tested for single-dose enzyme inhibition at a concentration of 20 μM. For the control compound, staurosporine was tested in a 10-dose IC_50_ mode. To determine their IC_50_ values, the compounds were tested at 10-concentrations with 3-fold serial dilution starting at 100 μM.

### 3.3. Molecular Docking Analysis

Molecular docking was performed using CDOCKER, Discovery Studio (DS) 2020 (Dassault Systèmes, BIOVIA, San Diego, CA, USA). The crystal structure of BTK (PDB ID: 3PJ3) [15] was obtained from the protein data bank. The docking parameters were set to default. The ligands and protein were prepared using the CHARMm forcefield. Water molecules were removed and hydrogen atoms were added during the preparation process. The active site was identified from the original ligand of 3PJ3 using the Define and Edit Binding Site tool. The missing loops of the 3PJ3 protein were not added back, and we set the CDOCKER parameters to give 10 diverse poses, and the pose cluster radius as 0.1 for precise docking results. The docking results were analyzed and visualized using Discovery Studio.

### 3.4. In Vitro Growth Inhibition

Cellular toxicity of the compounds was measured using the CellTiter-Glo luminescent cell viability assay (Promega, Madison, WI, USA). Raji (ATCC CCL-86) and Ramos (ATCC CRL-1596) cells were seeded on an opaque-walled 96-well plate (1 × 10^4^ cells/well) and treated with various concentrations of **17d** and **18g**. After 48 h, 100 µL of CellTiter-Glo Reagent (Promega, Madison, WI, USA) was added to each well and the plate was incubated at room temperature for 10 min. The luminescence signal was measured using a GloMax-Multi Detection System (Promega, Madison, WI, USA) and GI_50_ values were calculated using relative IC_50_ model in GraphPad Prism (GraphPad, San Diego, CA, USA).

### 3.5. In Vivo Xenograft Model

The animal study was approved by the Institutional Animal Care and Use Committee (IACUC) of Korea Research Institute of Bioscience and Biotechnology on 28 January 2020 and the approval number was registered as KRIBB-AEC-19277. Raji cells (9 × 10^6^ cells/mouse) were inoculated subcutaneously in the right flank of 6-week-old female BALB/c nu/nu mice (Orient Bio, Seongnam, Gyeonggi-do, Korea). After 1 week, the mice were randomly distributed between the control and treatment groups, and treated five times per week with vehicle (5% DMSO and 5% Tween 80 in corn oil), 2 mg/kg of doxorubicin hydrochloride, or 50 mg/kg of compound **17d** and **18g**. During treatment, tumor volumes were measured with vernier calipers and estimated by the formula: length (mm) × width (mm) × height (mm)/2, and body weight was monitored three times a week. On day 14, the mice were sacrificed and tumor weights were measured. Statistical analyses were carried out using GraphPad Prism (GraphPad, San Diego, CA, USA). Changes in body weight and tumor volume were analyzed using two-way ANOVA and Bonferroni multiple comparison test, and *p* values of <0.05 were considered statistically significant.

## 4. Conclusions

In summary, 5-phenoxy-2-aminopyridine derivatives were designed and synthesized as covalent BTK inhibitors and their in vitro and in vivo effects in experimental models of hematological malignancy were tested. Of the tested compounds, **18g** showed the highest in vitro enzyme inhibitory activity and best selectivity profile. As shown by molecular docking, introducing a tetrahydroisoquinoline linker directed the lipophilic moiety to occupy the allosteric pocket that was generated by the DFG-out conformation, which can provide novel perspectives for future structure-based drug discovery of a BTK inhibitors with a unique BTK binding mode. Further efforts will include corroboration of irreversible binding of the Michael acceptor-containing compounds by comparing their inhibitory activity against wild-type and mutant (C481S) BTK. Compound **18g** also showed good antiproliferative activity in both Raji and Ramos cell lines in vitro and the Raji xenograft mouse model in vivo. Therefore, we propose the novel 5-phenoxy-2-aminopyridine derivatives as potent and selective BTK inhibitors with the potential for further development as chemotherapeutic and anti-inflammatory agents.

## Figures and Tables

**Table 1:** Structure–activity relationship of piperazine-linked aminopyridine **6.**

Compound	R_1_	% Inhibition ^1^	IC_50_ (μM) ^2^
**6a**	H	14	ND ^3^
**6b**	Bn	85	3.17
**6c**	*p*-FBn	83	ND
**6d**	*p*-ClBn	92	1.54
**6e**	PhEt	89	5.01
**6f**	*p*-ClPhEt	52	ND
**6g**	benzyloxycarbonyl	77	ND
Ibrutinib			0.0002

^1^ Relative percentage inhibition at 20 μM of compound compared with staurosporine, HotSpot kinase assay (Reaction Biology Corp.); ^2^ HotSpot kinase assay (Reaction Biology Corp.); ^3^ ND = not determined.

**Table 2:** SAR of tetrahydroisoquinoline linked aminopyridines **17** and **18**.

Compound	Position	R_3_	% Inhibition ^1^	IC_50_ (μM) ^2^
**17a**	2	H	98	0.63
**17d**	2	OBn	98	0.46
**18a**	3	H	98	2.20
**18b**	3	O*^n^*Pr	99	0.97
**18c**	3	OCH_2_*^c^*Pr	96	1.20
**18d**	3	OBn	97	0.10
**18e**	3	O*p*-FBn	53 *	8.53
**18f**	3	O*p*-ClBn	98	1.00
**18g**	3	O*p*-OMeBn	92 *	0.19

^1^ Relative percentage inhibition at 20 μM of compound compared with staurosporine, ADP-Glo Assay (Promega), * HotSpot kinase assay (Reaction Biology Corp.); ^2^ ADP-Glo Assay (Promega). IC_50_ values were calculated using Sigmaplot software ver. 12.0.

**Table 3:** Kinase selectivity profiles of **17d**, **18d**, and **18g**.

Kinase	% Inhibition ^1^		
	17d	18d	18g
BMX/ETK	98.7 ± 0.6	95.5 ± 0.1	96.0 ± 0.1
EGFRK	87.0 ± 0.3	60.5 ± 4.4	33.5 ± 0.5
ERBB2	43.4 ± 1.0	82.3 ± 1.6	78.8 ± 0.7
ERBB4	95.0 ± 0.1	96.2 ± 0.7	95.0 ± 0.1
ITK	75.1 ± 0.0	52.8 ± 1.8	46.0 ± 2.3
JAK3	64.8 ± 0.3	46.2 ± 4.2	77.1 ± 3.0
TEC	84.1 ± 0.2	71.3 ± 1.0	66.4 ± 0.9
TXK	88.8 ± 0.1	37.5 ± 3.3	49.7 ± 2.5

^1^ Relative percentage inhibition at 20 μM of compound compared with staurosporine, HotSpot kinase assay (Reaction Biology Corp.). Compounds were tested in duplicate. Red, ≥90% inhibition; medium red, 80–90% inhibition; light pink, 60–80% inhibition; no color, <60% inhibition. Values are expressed as means ± standard deviation (SD): BMX/ETK, bone marrow X-linked kinase/epithelial and endothelial tyrosine kinase; EGFRK, epidermal growth factor receptor kinase; ERBB, erythroblastic leukemia viral oncogene homolog; ITK, interleukin-2-inducible T-cell kinase; JAK3, janus kinase 3; TEC, tyrosine protein kinase Tec; TXK, TXK tyrosine kinase.

**Table 4:** In vitro cell growth inhibitory activity of **17d** and **18g**.

Compound	GI_50_ (μM) ^1^			
	Raji ^2^	Ramos ^2^	MRC-5 ^3^	MCF10A ^3^
**17d**	16.1 ± 0.5	12.5 ± 0.1	28.4 ± 4.0	33.4 ± 2.2
**18g**	25.4 ± 1.4	16.2 ± 1.2	>100	>100
Ibrutinib	9.5 ± 0.2	6.3 ± 4.8	40.4 ± 0.9	4.6 ± 1.1

^1^ CellTiter-Glo assay (Promega). Values are expressed as means ± standard deviation (SD); ^2^ compounds **17d** and **18g** were tested with four replicates and ibrutinib was tested with six replicates; ^3^ compounds were tested with four replicates. GI_50_ values were calculated using GraphPad Prism: Raji and Ramos, human B Burkitt-lymphocyte lymphoma cell lines; MRC-5, human lung normal cell; MCF10A, non-malignant human breast cell line.

## Supplementary Materials

Supplementary Materials can be found at https://www.mdpi.com/1422-0067/21/21/8006/s1.

## Abbreviations

FDA	Food and Drug Administration
JAK3	Janus kinase 3
EGFRK	Epidermal growth factor receptor kinase
ATP	Adenosine triphosphate
SRC	Proto-oncogene tyrosine-protein kinase Src
Boc	*tert*-Butyloxycarbonyl protecting group
TFA	Trifluoroacetic acid
DCM	Dichloromethane
DMF	Dimethylformamide
TEA	Triethylamine
PPA	Polyphosphoric acid
THF	Tetrahydrofuran
DMSO	Dimethyl sulfoxide
ADP	Adenosine diphosphate
IC_50_	The half maximal inhibitory concentration
Bn	Benzyl
Me	Methyl
Et	Ethyl
Pr	Propyl
Ac	Acetyl
GI_50_	The half maximal growth inhibitory concentration
