# Supplementary material for: Discovery of 5-Phenoxy-2-aminopyridine Derivatives as Potent and Selective Irreversible Inhibitors of Bruton’s Tyrosine Kinase
Source: Int J Mol Sci. 2020 Oct 28;21(21):8006. doi: 10.3390/ijms21218006 (PMC7663149; doi:10.3390/ijms21218006)
Supplement: Supplementary file 1 [file ijms-21-08006-s001.pdf]

International Journal of
Molecular Sciences

MDPI

Supplementary Materials

1. Preparation of Compounds 1 to 16

Most reagents and solvents were purchased from commercial sources and used without further purification. Flash column chromatography was performed using silica gel 60 (Merck Millipore, Burlington, MA, USA, 230–400 mesh) with the indicated solvents. To monitor the completion of the reaction, thin-layer chromatography (TLC) was performed using Kieselgel 60 F254 plates (Merck Millipore, Burlington, MA, USA). IR spectra were recorded by a Nicolet iS5 spectrometer (Thermo Fisher Scientific, Waltham, MA, USA) using the ATR method. NMR spectra were recorded on an Avance III HD 500 spectrometer (Bruker, Billerica, MA, USA, ^{1}H 500 MHz, and ^{13}C 125 MHz). ^{19}F NMR spectra were not determined.

*1.1. 3,5-Dichloro-2-nitropyridine (**1**)*

To a mixture of potassium persulfate (8.3 g, 30.7 mmol) in sulfuric acid (30 ml) was added 3,5-dichloro-2-aminopyridine (1.0 g, 6.14 mmol). The mixture was stirred at 90 ˚C for 23 h. The mixture was neutralized with 50% NaOH solution and extracted with EtOAc. The combined organic layers were dried over $MgSO_4$, filtered and concentrated in vacuo. The residue was purified by flash column chromatography (*n*-hexane/EtOAc = 4:1) to provide **1** as a brown liquid (540 mg, 45%): IR (neat, cm^{-1}); 3065, 1571, 1542; ^{1}H NMR (500 MHz, $CDCl_3$) δ 8.41 (1H, d, *J* = 2.0 Hz), 8.03 (1H, d, *J* = 2.0 Hz); ^{13}C NMR (125 MHz, $CDCl_3$) δ 153.8, 145.5, 140.5, 136.0, 124.2.

*1.2. (5-Chloro-2-nitropyridin-3-yl)-piperazine (**2**)*

To a mixture of compound **1** (1.9 g, 9.66 mmol) in toluene (9.7 ml) was added K_2CO_3 (2.7 g, 19.3 mmol) under a nitrogen atmosphere. After stirring at rt for 10 min, 1-Boc-piperazine (1.8 g, 9.66 mmol) was added and heated at 50 ˚C for 15 h. After cooling to rt, the mixture was diluted with EtOAc, washed with water and brine. The combined organic layers were dried over $MgSO_4$, filtered and concentrated in vacuo. The residue was purified by flash column chromatography (*n*-hexane/EtOAc = 7:1) to provide intermediate 4-Boc-(5-chloro-2-nitropyridin-3-yl)piperazine as a brown liquid (1.4 g, 42%).

To a stirred solution of TFA (76 μl) in DCM (0.4 ml) was added intermediate 4-Boc-(5-chloro-2-nitropyridin-3-yl)piperazine (67 mg, 0.196 mmol) chilled to below 0 ˚C. The resulting mixture was stirred for 4 h at rt. The mixture was basified with $NaHCO_3$ (aq) to pH 7 and was extracted with DCM. The combined organic layers were dried over $MgSO_4$, filtered and concentrated in vacuo. The residue was purified by flash column chromatography ($CHCl_3$/MeOH = 10:1) to provide **2** as an orange solid (38 mg, 79%): ^{1}H NMR (500 MHz, $CDCl_3$) δ 7.98 (1H, d, *J* = 2.0 Hz), 7.48 (1H, d, *J* = 2.0 Hz), 3.07 (4H, dd, *J* = 3.0, 6.0 Hz), 3.01 (4H, dd, *J* = 3.0, 6.0 Hz).

*1.3. General Procedures for the Synthesis of Compounds **3a-G***

To a mixture of compound **2** (1.0 equiv.) in DMF was added K_2CO_3 (2.0 equiv.) under a nitrogen atmosphere. After stirring at rt for 10 min, the mixture was cooled in an ice bath and alkyl halide (1.5 equiv.) was added. The reaction mixture was stirred at rt for 5 h and diluted with EtOAc, washed with water and brine. The combined organic layers were dried over $MgSO_4$, filtered and concentrated in vacuo. The residue was purified by flash column chromatography (*n*-hexane/EtOAc) to provide **3**.

1.3.1. Benzyl-4-(5-chloro-2-nitropyridin-3-yl)piperazine (**3b**)

For the synthesis of **3b**, compound **2** (581 mg, 2.39 mmol) and benzyl bromide (620 mg, 3.59 mmol) were used: Yellow liquid (yield 77%); ^{1}H NMR (500 MHz, $CDCl_3$) δ 7.96 (1H, d, *J* = 2.0 Hz), 7.46 (1H, d, *J* = 2.0 Hz), 7.36-7.26 (5H, m), 3.57 (2H, s), 3.11 (4H, t, *J* = 5.0 Hz), 2.59 (4H, t, *J* = 5.0 Hz).

1.3.2. (5-Chloro-2-nitropyridin-3-yl)-4-(4-fluorobenzyl)piperazine (**3c**)

For the synthesis of **3c**, compound **2** (30 mg, 0.122 mmol) and 4-fluorobenzyl bromide (35 mg, 0.183 mmol) were used: Yellow liquid (yield 73%); ^{1}H NMR (500 MHz, $CDCl_3$) δ 7.96 (1H, d, *J* = 2.0 Hz), 7.47 (1H, d, *J* = 2.0 Hz), 7.29 (2H, dd, *J* = 5.5, 8.5 Hz), 7.01 (2H, t, *J* = 8.5 Hz), 3.53 (2H, s), 3.10 (4H, t, *J* = 5.0 Hz), 2.57 (4H, t, *J* = 5.0 Hz).

1.3.3. (4-Chlorobenzyl)-4-(5-chloro-2-nitropyridin-3-yl)piperazine (**3d**)

For the synthesis of **3d**, compound **2** (40 mg, 0.165 mmol) and 4-chlorobenzyl chloride (40 mg, 0.248 mmol) were used: Yellow liquid (yield 92%); ^{1}H NMR (500 MHz, $CDCl_3$) δ 7.97 (1H, d, *J* = 2.0 Hz), 7.47 (1H, d, *J* = 2.0 Hz), 7.31-7.25 (4H, m), 3.52 (2H, s), 3.10 (4H, t, *J* = 5.0 Hz), 2.57 (4H, t, *J* = 5.0 Hz).

1.3.4. (5-Chloro-2-nitropyridin-3-yl)-4-phenethylpiperazine (**3e**)

For the synthesis of **3e**, compound **2** (629 mg, 2.59 mmol) and 2-bromoethyl benzene (734 mg, 3.89 mmol) were used: Yellow liquid (yield 40%); ^{1}H NMR (500 MHz, $CDCl_3$) δ 7.93 (1H, d, *J* = 2.0 Hz), 7.49 (1H, d, *J* = 2.0 Hz), 7.27 (2H, t, *J* = 8.0 Hz), 7.22-7.16 (3H, m), 3.11 (4H, t, *J* = 5.0 Hz), 2.82-2.79 (2H, m), 2.67-2.64 (6H, m).

1.3.5. (5-Chloro-2-nitropyridin-3-yl)-4-(4-chlorophenethyl)piperazine (**3f**)

For the synthesis of **3f**, compound **2** (143 mg, 0.590 mmol) and 4-chlorophenethyl bromide (200 mg, 0.885 mmol) were used: Yellow liquid (yield 53%); ^{1}H NMR (500 MHz, $CDCl_3$) δ 7.97 (1H, d, *J* = 2.0 Hz), 7.49 (1H, d, *J* = 2.0 Hz), 7.25 (2H, d, *J* = 8.5 Hz), 7.13 (2H, d, *J* = 8.5 Hz), 3.13 (4H, t, *J* = 5.0 Hz), 2.80-2.76 (2H, m), 2.66-2.63 (6H, m).

1.3.6. 4-(5-Chloro-2-nitropyridin-3-yl)piperazine Carboxylic Acid Benzyl Ester (**3g**)

For the synthesis of **3g**, compound **2** (266 mg, 1.10 mmol) and benzyl chloroformate (293 mg, 1.65 mmol) were used: Yellow liquid (yield 93%); ^{1}H NMR (500 MHz, $CDCl_3$) δ 8.05 (1H, d, *J* = 2.0 Hz), 7.50 (1H, d, *J* = 2.0 Hz), 7.39-7.33 (5H, m), 5.16 (2H, s), 3.66 (4H, t, *J* = 5.0 Hz), 3.07 (4H, br s).

1.4. General Procedures for the Synthesis of Compounds ***4a-g***

To a mixture of compound **3** (1.0 equiv.) in DMF was added Cs_2CO_3 (2.0 equiv.) under a nitrogen atmosphere. After stirring at rt for 10 min, resorcinol (1.0 equiv.) was added and the mixture was heated at 70 °C for 24 h. After cooling to rt, the mixture was diluted with EtOAc, washed with water and brine. The combined organic layers were dried over $MgSO_4$, filtered and concentrated in vacuo. The residue was purified by flash column chromatography (*n*-hexane/EtOAc) to provide **4**.

1.4.1. (6-Nitro-5-piperazinylpyridin-3-yloxy)phen-3-ol (**4a**)

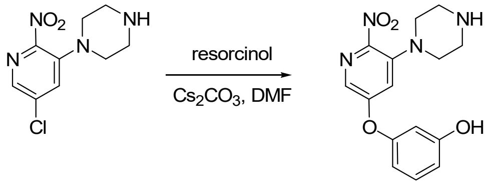

To a mixture of compound **2** (597 mg, 2.46 mmol) in DMF (12.3 ml) was added Cs_2CO_3 (1.2 g, 3.69 mmol) under a nitrogen atmosphere. After stirring at rt for 10 min, resorcinol (271 mg, 2.46 mmol) was added and the mixture was heated at 70 °C for 24 h. After cooling to rt, the mixture was diluted with EtOAc, washed with water and brine. The combined organic layers were dried over $MgSO_4$, filtered and concentrated in vacuo. The residue was purified by flash column chromatography (*n*-hexane/EtOAc/MeOH = 2:1:0.1) to provide **4a** as a yellow liquid (152 mg, 20%): ^{1}H NMR (500 MHz,

$CDCl_3$) δ 7.72 (1H, d, *J* = 2.0 Hz), 7.22 (1H, t, *J* = 8.0 Hz), 7.17 (1H, d, *J* = 2.0 Hz), 6.64 (2H, dd, *J* = 2.0, 8.0 Hz), 6.54 (1H, t, *J* = 2.0 Hz), 3.15 (4H, br s), 3.06 (4H, br s).

1.4.2. 5-(4-Benzylpiperazinyl)-6-nitropyridin-3-yloxyphen-3-ol (**4b**)

Yellow liquid (yield 79%); ^{1}H NMR (500 MHz, $CDCl_3$) δ 7.74 (1H, d, *J* = 2.5 Hz), 7.33-7.26 (5H, m), 7.21 (1H, t, *J* = 8.0 Hz), 6.98 (1H, d, *J* = 2.5 Hz), 6.61 (1H, dd, *J* = 2.0, 8.0 Hz), 6.59 (1H, dd, *J* = 2.0, 8.0 Hz), 6.50 (2H, t, *J* = 2.0 Hz), 3.59 (2H, s), 3.05 (4H, t, *J* = 5.0 Hz), 2.63 (4H, t, *J* = 5.0 Hz).

1.4.3. 5-[4-(4-Fluorobenzyl)piperazinyl]-6-nitropyridin-3-yloxyphen-3-ol (**4c**)

Yellow liquid (yield 76%); ^{1}H NMR (500 MHz, $CDCl_3$) δ 7.73 (1H, d, *J* = 2.5 Hz), 7.29-7.26 (2H, m), 7.22 (1H, t, *J* = 8.0 Hz), 7.01-6.98 (3H, m), 6.62 (1H, dd, *J* = 2.0, 8.5 Hz), 6.59 (1H, dd, *J* = 2.0, 8.0 Hz), 6.50 (1H, t, *J* = 2.0 Hz), 3.54 (2H, s), 3.04 (4H, t, *J* = 5.0 Hz), 2.60 (4H, t, *J* = 5.0 Hz).

1.4.4. 5-[4-(4-Chlorobenzyl)piperazinyl]-6-nitropyridin-3-yloxyphen-3-ol (**4d**)

Yellow liquid (yield 78%); ^{1}H NMR (500 MHz, $CDCl_3$) δ 7.74 (1H, d, *J* = 2.5 Hz), 7.29-7.27 (2H, m), 7.25-7.22 (3H, m), 6.99 (1H, d, *J* = 2.5 Hz), 6.66 (1H, dd, *J* = 2.0, 8.0 Hz), 6.60 (1H, dd, *J* = 2.0, 8.0 Hz), 6.53 (1H, t, *J* = 2.0 Hz), 3.53 (2H, s), 3.04 (4H, t, *J* = 5.0 Hz), 2.59 (4H, t, *J* = 5.0 Hz).

1.4.5. 6-Nitro-5-(4-phenethylpiperazinyl)pyridin-3-yloxyphen-3-ol (**4e**)

Yellow liquid (yield 67 %); ^{1}H NMR (500 MHz, $CDCl_3$) δ 7.79 (1H, d, *J* = 2.5 Hz), 7.30-7.17 (6H, m), 7.00 (1H, d, *J* = 2.5 Hz), 6.65 (1H, dd, *J* = 2.0, 8.0 Hz), 6.61 (1H, dd, *J* = 2.0, 8.0 Hz), 6.54 (1H, t, *J* = 2.0 Hz), 3.09 (4H, t, *J* = 5.0 Hz), 2.85-2.82 (2H, m), 2.74-2.70 (6H, m).

1.4.6. 6-Nitro-5-[4-(4-chlorophenethyl)piperazinyl]pyridin-3-yloxyphen-3-ol (**4f**)

Yellow liquid (yield 56%); ^{1}H NMR (500 MHz, $CDCl_3$) δ 7.77 (1H, d, *J* = 2.5 Hz), 7.27-7.24 (3H, m), 7.11 (2H, d, *J* = 8.0 Hz), 7.01 (1H, d, *J* = 2.5 Hz), 6.68 (1H, dd, *J* = 2.0, 8.0 Hz), 6.61 (1H, dd, *J* = 2.0, 8.0 Hz), 6.56 (1H, t, *J* = 2.0 Hz), 3.07 (4H, t, *J* = 5.0 Hz), 2.80-2.77 (2H, m), 2.69-2.64 (6H, m).

1.4.7. 4-[5-(3-Hydroxyphenoxy)-2-nitropyridin-3-yl]piperazine carboxylic acid benzyl Ester (**4g**)

Yellow liquid (yield 52%); ^{1}H NMR (500 MHz, $CDCl_3$) δ 7.72 (1H, d, *J* = 2.0 Hz), 7.37-7.32 (5H, m), 7.20 (1H, t, *J* = 8.0 Hz), 7.16 (1H, d, *J* = 2.0 Hz), 6.61 (2H, t, *J* = 8.5 Hz), 6.52 (1H, s), 6.40 (1H, br s), 5.16 (2H, s), 3.66 (4H, t, *J* = 5.0 Hz), 3.12 (4H, br s).

*1.5. General Procedures for the Synthesis of Compounds **5a-g***

To a mixture of compound **4** (1.0 equiv.) in DCM was added TEA (1.0 equiv.) under a nitrogen atmosphere. The mixture was stirred at rt for 10 min, and then the mixture was cooled in an ice bath and acryloyl chloride (2.0 equiv.) was added. After stirring for 1 h, the reaction mixture was neutralized with $NaHCO_3$ (aq) and extracted with DCM. The combined organic layers were dried over $MgSO_4$, filtered and concentrated in vacuo. The residue was purified by flash column chromatography ($CHCl_3$/EtOAc) to provide **5**.

1.5.1. Acrylic Acid 3-(6-nitro-5-piperazinylpyridin-3-yloxy)phenyl Ester (**5a**)

Yellow liquid (yield 6%); ^{1}H NMR (500 MHz, $CDCl_3$) δ 7.84 (1H, d, *J* = 2.5 Hz), 7.28 (1H, t, *J* = 8.0 Hz), 7.01 (1H, d, *J* = 2.5 Hz), 6.74 (1H, dd, *J* = 2.5, 8.0 Hz), 6.64 (1H, dd, *J* = 2.5, 8.0 Hz), 6.59 (1H, t, *J* = 2.5 Hz), 6.55 (1H, dd, *J* = 10.5, 17.0 Hz), 6.33 (1H, dd, *J* =1.5, 17.0 Hz), 5.75 (1H, dd, *J* = 1.5, 10.5 Hz), 5.42 (1H, br s), 3.84 (2H, br s), 3.72 (2H, br s), 3.06 (4H, br s).

1.5.2. Acrylic Acid 3-[5-(4-benzylpiperazinyl)-6-nitropyridin-3-yloxy]phenyl Ester (**5b**)

Yellow liquid (yield 59%); ^1H NMR (500 MHz, $CDCl_3$) δ 7.77 (1H, d, *J* = 2.0 Hz), 7.43 (1H, t, *J* = 8.0 Hz), 7.34-7.24 (5H, m), 7.04-7.02 (2H, m), 6.97 (1H, dd, *J* = 2.5, 8.5 Hz), 6.91 (1H, t, *J* = 2.5 Hz), 6.61 (1H, dd, *J* = 1.0, 17.5 Hz), 6.30 (1H, dd, *J* = 10.5, 17.5 Hz), 6.04 (1H, dd, *J* = 1.0, 10.5 Hz), 3.55 (2H, s), 3.06 (4H, t, *J* = 5.0 Hz), 2.58 (4H, t, *J* = 5.0 Hz).

1.5.3. Acrylic Acid 3-{5-[4-(4-fluorobenzyl)piperazinyl]-6-nitropyridin-3-yloxy}phenyl Ester (**5c**)

Yellow liquid (yield 51%); ^1H NMR (500 MHz, $CDCl_3$) δ 7.77 (1H, d, *J* = 2.5 Hz), 7.43 (1H, t, *J* = 8.0 Hz), 7.27 (2H, dd, *J* = 5.5, 8.0 Hz), 7.04-6.96 (5H, m), 6.91 (1H, t, *J* = 2.0 Hz), 6.60 (1H, dd, *J* = 1.0, 17.5 Hz), 6.30 (1H, dd, *J* = 10.5, 17.5 Hz), 6.04 (1H, dd, *J* = 1.0, 10.5 Hz), 3.50 (2H, s), 3.05 (4H, t, *J* = 5.0 Hz), 2.55 (4H, t, *J* = 5.0 Hz).

1.5.4. Acrylic Acid 3-{5-[4-(4-chlorobenzyl)piperazinyl]-6-nitropyridin-3-yloxy}phenyl Ester (**5d**)

Yellow liquid (yield 51%); ^1H NMR (500 MHz, $CDCl_3$) δ 7.78 (1H, d, *J* = 2.5 Hz), 7.43 (1H, t, *J* = 8.0 Hz), 7.29-7.24 (4H, m), 7.04-7.02 (2H, m), 6.97 (1H, dd, *J* = 2.0, 8.0 Hz), 6.91 (1H, t, *J* = 2.0 Hz), 6.61 (1H, dd, *J* = 1.0, 17.5 Hz), 6.30 (1H, dd, *J* = 10.5, 17.5 Hz), 6.05 (1H, dd, *J* = 1.0, 10.5 Hz), 3.51 (2H, s), 3.05 (4H, t, *J* = 5.0 Hz), 2.56 (4H, t, *J* = 5.0 Hz).

1.5.5. Acrylic Acid 3-[6-nitro-5-(4-phenethylpiperazinyl)pyridin-3-yloxy]phenyl Ester (**5e**)

Yellow liquid (yield 67%); ^1H NMR (500 MHz, $CDCl_3$) δ 7.79 (1H, d, *J* = 2.5 Hz), 7.45 (1H, t, *J* = 8.5 Hz), 7.29 (2H, t, *J* = 8.0 Hz), 7.22-7.19 (3H, m), 7.07 (1H, d, *J* = 2.0 Hz), 7.04 (1H, dd, *J* = 2.0, 8.0 Hz), 6.98 (1H, dd, *J* = 2.0, 8.0 Hz), 6.92 (1H, t, *J* = 2.0 Hz), 6.61 (1H, dd, *J* = 1.0, 17.5 Hz), 6.31 (1H, dd, *J* = 10.5, 17.5 Hz), 6.05 (1H, dd, *J* = 1.0, 10.5 Hz), 3.11 (4H, t, *J* = 5.0 Hz), 2.84-2.81 (2H, m), 2.71-2.67 (6H, m).

1.5.6. Acrylic Acid 3-{6-nitro-5-[4-(4-chlorophenethyl)piperazinyl]pyridin-3-yloxy}phenyl Ester (**5f**)

Yellow liquid (yield 48%); ^1H NMR (500 MHz, $CDCl_3$) δ 7.79 (1H, d, *J* = 2.5 Hz), 7.44 (1H, t, *J* = 8.0 Hz), 7.25 (2H, d, *J* = 8.5 Hz), 7.13 (2H, d, *J* = 8.5 Hz), 7.06 (1H, d, *J* = 2.5 Hz), 7.04 (1H, dd, *J* = 2.0, 8.0 Hz), 6.98 (1H, dd, *J* = 2.0, 8.0 Hz), 6.92 (1H, t, *J* = 2.0 Hz), 6.61 (1H, dd, *J* = 1.0, 17.5 Hz), 6.31 (1H, dd, *J* = 10.5, 17.5 Hz), 6.05 (1H, dd, *J* = 1.0, 10.5 Hz), 3.08 (4H, t, *J* = 5.0 Hz), 2.78-2.75 (2H, m), 2.65-2.61 (6H, m).

1.5.7. 4-[5-(3-Acryloyloxyphenoxy)-2-nitropyridin-3-yl]piperazine carboxylic acid benzyl Ester (**5g**)

Yellow liquid (yield 62%); ^1H NMR (500 MHz, $CDCl_3$) δ 7.85 (1H, d, *J* = 2.0 Hz), 7.45 (1H, t, *J* = 8.0 Hz), 7.38-7.33 (5H, m), 7.05-7.04 (2H, m), 6.99 (1H, dd, *J* = 2.0, 8.5 Hz), 6.92 (1H, s), 6.61 (1H, d, *J* = 17.5 Hz), 6.31 (1H, dd, *J* = 10.5, 17.5 Hz), 6.05 (1H, d, *J* = 10.5 Hz), 5.15 (2H, s), 3.65 (4H, t, *J* = 5.0 Hz), 3.02 (4H, br s).

*1.6. 2-(3-Methoxyphenyl)ethyl Carbamic Acid Ethyl Ester (**7**)*

To a mixture of 2-(3-methoxyphenyl)ethylamine (2.0 g, 13.2 mmol) in DCM (13 ml) under nitrogen atmosphere was added TEA (3.7 ml, 26.5 mmol) at rt. After stirring for 10 min ethyl chloroformate (1.0 g, 26.5 mmol) was added and the reaction mixture was stirred for 2 h. The reaction mixture was diluted with EtOAc, washed with water and brine, and dried over $MgSO_4$, filtered and concentrated in vacuo. The residue was filtered with diethyl ether to provide **7** as a yellow liquid without further purification (1.7 g, 13.2 mmol): IR (neat, cm^{-1}); 2980, 2926, 1701, 1584, 1524, 1446, 1251, 1237, 1151, 1039, 763; ^1H NMR (500 MHz, $CDCl_3$) δ 7.22 (1H, t, *J* = 8.0 Hz), 6.79-6.76 (2H, m), 6.74 (1H, s), 4.67 (1H, br s), 4.10 (2H, q, *J* = 7.0 Hz), 3.80 (3H, s), 3.43 (2H, t, *J* = 7.0 Hz), 2.79 (2H, t, *J* = 7.0 Hz), 1.23 (3H, t, *J* = 7.0 Hz); ^{13}C NMR (125 MHz, $CDCl_3$) δ 159.8, 156.3, 140.5, 129.6, 121.1, 114.5, 111.8, 60.7, 55.1, 42.0, 36.2, 14.6.

*1.7. 6-Methoxy-3,4-dihydro-2H-isoquinolinone (**8**)*

To compound **7** (1.7 g, 7.40 mmol) was added polyphosphoric acid (5.8 g, 59.2 mmol) at 120 °C. After stirring for 1.5 h, the reaction mixture was neutralized with $NaHCO_3$ (aq) and extracted with EtOAc. The combined organic layers were dried over $MgSO_4$, filtered and concentrated in vacuo. The residue was purified by flash column chromatography (*n*-hexane/EtOAc = 1:10) to provide **8** as a white solid (889 mg, 68%): IR (neat, cm^{-1}); 3195, 3064, 1647, 1604, 1477, 1460, 1269, 1251, 1025, 802; ^1H NMR (500 MHz,$CDCl_3$) δ 8.01 (1H, d, *J* = 8.5 Hz), 6.86 (1H, dd, *J* = 2.5, 8.5 Hz), 6.71 (1H, d, *J* = 2.5 Hz), 6.58 (1H, br s), 3.85 (3H, s), 3.56 (2H, td, *J* = 3.0, 6.5 Hz), 2.97 (2H, t, *J* = 6.5Hz); ^{13}C NMR (125 MHz, $CDCl_3$) δ 166.8, 162.6, 141.1, 130.1, 121.9, 112.5, 112.4, 55.5, 40.3, 28.8.

*1.8. 6-Hydroxy-3,4-dihydro-2H-isoquinolinone (**9**)*

To compound **8** (414 mg, 2.34 mmol) was added 48% hydrobromic acid (8 ml) and the mixture was refluxed for 24 h. The mixture was neutralized with $NaHCO_3$ (aq) and extracted with EtOAc. The combined organic layers were dried over $MgSO_4$, filtered and concentrated in vacuo. The residue was purified by flash column chromatography (DCM/MeOH = 10:1) to provide **9** as a white solid (198 mg, 63%): IR (neat, cm^{-1}); 3303, 3071 2982, 2938, 2835, 1596, 1465; ^1H NMR (500 MHz, CD_3OD) δ 7.78 (1H, d, *J* = 8.0 Hz), 6.73 (1H, dd, *J* = 2.5, 8.0 Hz), 6.65 (1H, d, *J* = 2.5 Hz), 3.46 (2H, t, *J* = 6.5 Hz), 2.88 (2H, t, *J* = 6.5 Hz); ^{13}C NMR (125 MHz, CD_3OD) δ 168.8, 162.8, 143.2, 130.8, 121.2, 115.1, 114.7, 40.9, 29.3.

*1.9. General Procedures for the Synthesis of Compounds **10a-g***

To a mixture of compound **9** (1.0 equiv.) in DMF was added K_2CO_3 (2.0 equiv.) under a nitrogen atmosphere. After stirring at rt for 30 min, alkyl halide (1.5 equiv.) was added and the mixture heated at 60 °C for 4 h. After cooling to rt, the mixture was diluted with EtOAc, and washed with water and brine. The combined organic layers were dried over $MgSO_4$, filtered and concentrated under reduced pressure and then poured over diethyl ether. The precipitate was collected by filtration and the residual mixture was purified by flash column chromatography (*n*-hexane/EtOAc) to provide compound **10**.

1.9.1. 6-Propoxy-3,4-dihydro-*2H*-isoquinolinone (**10b**)

For the synthesis of **10b**, compound **9** (2.0 g, 12.3 mmol) and iodopropane (4.2 g, 24.5 mmol) were used: White solid (yield 55%); ^1H NMR (500 MHz, $CDCl_3$) δ 8.00 (1H, d, *J* = 8.5 Hz), 6.84 (1H, dd, *J* = 2.5, 8.5 Hz), 6.70 (1H, d, *J* = 2.5 Hz), 6.07 (1H, br s), 3.96 (2H, t, *J* = 6.5 Hz), 3.56 (2H, td, *J* = 3.0, 6.5 Hz), 2.96 (2H, t, *J* = 6.5 Hz), 1.82 (2H, sext., *J* = 7.0 Hz), 1.04 (3H, t, *J* = 7.0 Hz); ^{13}C NMR (125 MHz, $CDCl_3$) δ 166.5, 162.4, 141.0, 130.3, 121.6, 113.1, 113.0, 69.8, 40.5, 28.9, 22.6, 10.6.

1.9.2. 6-Cyclopropylmethoxy-3,4-dihydro-*2H*-isoquinolinone (**10c**)

For the synthesis of **10c**, compound **9** (2.0 g, 12.3 mmol) and (bromomethyl)cyclopropane (3.3 g, 24.5 mmol) were used: White solid (yield 52%); ^1H NMR (500 MHz, $CDCl_3$) δ 7.99 (1H, d, *J* = 8.5 Hz), 6.84 (1H, dd, *J* = 2.5, 8.5 Hz), 6.70 (1H, d, *J* = 2.5 Hz), 6.52 (1H, br s), 3.85 (1H, d, *J* = 7.0 Hz), 3.55 (2H, td, *J* = 3.0, 6.5 Hz), 2.95 (2H, t, *J* = 6.5 Hz), 1.31-1.25 (1H, m), 0.66 (2H, dd, *J* = 6.0, 13.5 Hz), 0.36 (2H, dd, *J* = 6.0, 10.5 Hz); ^{13}C NMR (125 MHz, $CDCl_3$) δ 166.9, 161.9, 141.0, 129.8, 121.6, 122.9, 122.8, 72.7, 40.0, 28.6, 10.1, 3.2.

1.9.3. 6-Benzyloxy-3,4-dihydro-*2H*-isoquinolinone (**10d**)

For the synthesis of **10d**, compound **9** (802 mg, 4.91 mmol) and benzyl bromide (1.3 g, 7.37 mmol) were used: White solid (yield 52%); ^1H NMR (500 MHz, $CDCl_3$) δ 8.01 (1H, d, *J* = 8.5 Hz), 7.43-7.38 (4H, m), 7.34 (1H, t, *J* = 7.0 Hz), 6.93 (1H, dd, *J* = 2.5, 8.5Hz), 6.84 (1H, br s), 6.78 (1H, d, *J* = 2.5 Hz), 5.10 (2H, s), 3.54 (2H, td, *J* = 3.0, 6.5 Hz), 2.94 (2H, t, *J* = 6.5 Hz).

1.9.4. 6-(4-Fluorobenzyloxy)-3,4-dihydro-*2H*-isoquinolinone (**10e**)

For the synthesis of **10e**, compound **9** (1.1 g, 6.68 mmol) and 4-fluorobenzyl chloride (1.9 g, 13.4 mmol) were used: White solid (yield 27%): ^{1}H NMR (500 MHz, $CDCl_3$) δ 8.02 (1H, d, *J* = 8.5Hz), 7.40 (2H, dd, *J* = 5.5, 8.5 Hz), 7.08 (2H, t, *J* = 8.5 Hz), 6.92 (1H, dd, *J* = 2.0, 8.5 Hz), 6.77 (1H, d, *J* = 2.0 Hz), 5.06 (2H, s), 3.55 (2H, td, *J* = 2.5, 6.5 Hz), 2.96 (2H, t, *J* = 6.5 Hz).

1.9.5. 6-(4-Chlorobenzyloxy)-3,4-dihydro-2*H*-isoquinolinone (**10f**)

For the synthesis of **10f**, compound **9** (1.1 g, 6.64 mmol) and 4-chlorobenzyl bromide (2.1 g, 13.3 mmol) were used: White solid (yield 52%); ^{1}H NMR (500 MHz, $CDCl_3$) δ 8.02 (1H, d, *J* = 8.5 Hz), 7.36 (4H, s), 6.91 (1H, dd, *J* = 2.5, 8.5 Hz), 6.76 (1H, d, *J* = 2.5 Hz), 6.17 (1H, br s), 5.07 (2H, s), 3.55 (2H, td, *J* = 3.0, 6.5 Hz), 2.96 (2H, t, *J* = 6.5 Hz).

1.9.6. 6-(4-Methoxybenzyloxy)-3,4-dihydro-2*H*-isoquinolinone (**10g**)

For the synthesis of **10g**, compound **9** (1.1 g, 6.74 mmol) and 4-methoxybenzyl chloride (2.1 g, 13.5 mmol) were used: White solid (yield 45%); ^{1}H NMR (500 MHz, $CDCl_3$) δ 8.19 (1H, d, *J* = 8.5 Hz), 7.35 (2H, d, *J* = 8.5 Hz), 6.94-6.92 (3H, m), 6.77 (1H, d, *J* = 2.5 Hz), 5.75 (1H, br s), 5.03 (2H, s), 3.83 (3H, s), 3.55 (2H, td, *J* = 3.0, 6.5 Hz), 2.96 (2H, t, *J* = 6.5 Hz).

*1.10. General Procedures for the Synthesis of Compounds **11a-g***

To a suspension of $LiAlH_4$ (2.0 equiv.) in dried THF was added compound **10** (1.0 equiv.) portion-wise at 0 °C. The reaction mixture was heated to reflux for 5 h. After cooling to rt, the mixture was added to water and Rochelle's salt, and was stirred for 2 h. The reaction mixture was diluted with EtOAc, washed with water and brine, and dried over $MgSO_4$, filtered and concentrated in vacuo. The collected compound **11** was used without further purification.

1.10.1. 6-Propoxy-1,2,3,4-tetrahydroisoquinoline (**11b**)

White solid (yield 50%); ^{1}H NMR (500 MHz, $CDCl_3$) δ 6.94 (1H, d, *J* = 8.5 Hz), 6.74 (1H, dd, *J* = 2.0, 8.5 Hz), 6.64 (1H, d, *J* = 2.0 Hz), 4.13 (1H, br s), 3.90 (2H, t, *J* = 6.5 Hz), 3.25 (2H, m), 2.90 (2H, m), 1.82 (2H, m), 1.04 (3H, t, *J* = 6.5 Hz).

1.10.2. 6-Cyclopropylmethoxy-1,2,3,4-tetrahydroisoquinoline (**11c**)

White solid (yield 40%); ^{1}H NMR (500 MHz, $CDCl_3$) δ 6.92 (1H, d, *J* = 8.5 Hz), 6.72 (1H, dd, *J* = 2.0, 8.5 Hz), 6.62 (1H, d, *J* = 2.0 Hz), 5.35 (1H, br s), 4.00 (2H, s), 3.76 (1H, d, *J* = 7.0 Hz), 3.166 (2H, s), 2.82 (2H, s), 1.28-1.23 (1H, m), 0.63 (2H, dd, *J* = 5.0, 13.0 Hz), 0.33 (2H, dd, *J* = 5.0, 10.5 Hz).

1.10.3. 6-Benzyloxy-1,2,3,4-tetrahydroisoquinoline (**11d**)

White solid (yield 84%); ^{1}H NMR (500 MHz, $CDCl_3$) δ 7.41 (2H, d, *J* = 7.5 Hz), 7.37 (2H, t, *J* = 7.5 Hz), 7.31 (1H, t, *J* = 7.5 Hz), 6.92 (1H, d, *J* = 8.5 Hz), 6.78 (1H, dd, *J* = 2.5, 8.5 Hz), 6.70 (1H, d, *J* = 2.5 Hz), 5.01 (2H, s), 4.71 (1H, br s), 4.00 (2H, s), 3.16 (2H, br s), 2.82 (2H, br s).

1.10.4. 6-(4-Fluorobenzyloxy)-1,2,3,4-tetrahydroisoquinoline (**11e**)

White solid (yield 95%); ^{1}H NMR (500 MHz, $CDCl_3$) δ 7.38 (2H, dd, *J* = 5.5, 8.0 Hz), 7.06 (2H, t, *J* = 8.0 Hz), 6.95 (1H, d, *J* = 8.0 Hz), 6.78 (1H, dd, *J* = 2.0, 8.0 Hz), 6.70 (1H, d, *J* = 2.0 Hz), 5.72 (1H, br s), 4.98 (2H, s), 4.04 (2H, s), 3.20 (2H, t, *J* = 6.0 Hz), 2.88 (2H, t, *J* = 6.0 Hz).

1.10.5. 6-(4-Chlorobenzyloxy)-1,2,3,4-tetrahydroisoquinoline (**11f**)

White solid (yield 88%); ^{1}H NMR (500 MHz, $CDCl_3$) δ 7.34 (4H, s), 6.92 (1H, d, *J* = 8.5 Hz), 6.75 (1H, dd, *J* = 2.5, 8.5 Hz), 6.68 (1H, d, *J* = 2.5 Hz), 4.98 (2H, s), 4.79 (1H, br s), 3.98 (2H, s), 3.14 (2H, t, *J* = 6.0 Hz), 2.80 (2H, t, *J* = 6.0 Hz).

1.10.6. 6-(4-Methoxybenzyloxy)-1,2,3,4-tetrahydroisoquinoline (**11g**)

White solid (yield 95%); ^{1}H NMR (500 MHz, $CDCl_3$) δ 7.33 (2H, d, *J* = 8.5 Hz), 6.91-6.89 (3H, m), 6.75 (1H, dd, *J* = 2.5, 8.0 Hz), 6.69 (1H, d, *J* = 2.5 Hz), 4.93 (2H, s), 3.93 (2H, s), 3.79 (3H, s), 3.10 (2H, t, *J* = 6.0 Hz), 2.92 (1H, br s), 2.75 (2H, t, *J* = 6.0 Hz).

1.11. General Procedures for the Synthesis of Compounds **12**

To a mixture of tetrahydroquinoline or compound **11** (1.0 equiv.) and K_2CO_3 (2.0 equiv.) in toluene was injected a solution of compound **1** (1.0 equiv.) in toluene under a nitrogen atmosphere. The reaction mixture was heated at 60 °C for 10 h. After cooling to rt, the mixture was diluted with EtOAc, washed with water and brine. The combined organic layers were dried over $MgSO_4$, filtered and concentrated in vacuo. The residue was purified by flash column chromatography (*n*-hexane/EtOAc) to provide compound **12**.

1.11.1. 2-(5-Chloro-2-nitropyridin-3-yl)-1,2,3,4-tetrahydroisoquinoline (**12a**)

Yellow solid (yield 34%); ^{1}H NMR (500 MHz, $CDCl_3$) δ 7.91 (1H, d, *J* = 2.0 Hz), 7.54 (1H, d, *J* = 2.0 Hz), 7.24-7.18 (3H, m), 7.12-7.10 (1H, m), 4.31 (2H, s), 3.44 (2H, t, *J* = 5.5 Hz), 3.00 (2H, t, *J* = 5.5 Hz).

1.11.2. 2-(5-Chloro-2-nitropyridin-3-yl)-6-propoxy-1,2,3,4-tetrahydroisoquinoline (**12b**)

Yellow solid (yield 57%); ^{1}H NMR (500 MHz, $CDCl_3$) δ 7.90 (1H, d, *J* = 2.0 Hz), 7.52 (1H, d, *J* = 2.0 Hz), 7.01 (1H, d, *J* = 8.5 Hz), 6.77 (1H, dd, *J* = 2.5, 8.5 Hz), 6.72 (1H, d, *J* = 2.5 Hz), 4.25 (2H, s), 3.91 (2H, t, *J* = 7.0 Hz), 3.40 (2H, t, *J* = 5.5Hz), 2.95 (2H, t, *J* = 5.5Hz), 1.80 (2H, sext., *J* = 7.0 Hz), 1.04 (3H, t, *J* = 7.0 Hz).

1.11.3. 2-(5-Chloro-2-nitropyridin-3-yl)-6-cyclopropylmethoxy-1,2,3,4-tetrahydroisoquinoline (**12c**)

Yellow solid (yield 43%); ^{1}H NMR (500 MHz, $CDCl_3$) δ 7.90 (1H, d, *J* = 1.5 Hz), 7.52 (1H, d, *J* = 2.0 Hz), 7.01 (1H, d, *J* = 8.5 Hz), 6.78 (1H, dd, *J* = 2.5, 8.5 Hz), 6.72 (1H, d, *J* = 2.5 Hz), 4.25 (2H,s), 3.79 (2H, d, *J* = 7.0 Hz), 3.40 (2H, t, *J* = 6.0 Hz), 2.95 (2H, t, *J* = 6.0 Hz), 1.29-1.24 (1H, m), 0.67-0.63 (2H, m), 0.35 (2H, dd, *J* = 5.0, 10.5 Hz).

1.11.4. 6-Benzyloxy-2-(5-chloro-2-nitropyridin-3-yl)-1,2,3,4-tetrahydroisoquinoline (**12d**)

Yellow solid (yield 40%); ^{1}H NMR (500 MHz, $CDCl_3$) δ 7.89 (1H, d, *J* = 2.0 Hz), 7.51 (1H, d, *J* = 2.0 Hz), 7.42 (2H, d, *J* = 7.0 Hz), 7.38 (2H, t, *J* = 7.0 Hz), 7.32 (1H, t, *J* = 7.0 Hz), 7.01 (1H, d, *J* = 8.5 Hz), 6.84 (1H, dd, *J* = 2.5, 8.5 Hz), 6.79 (1H, d, *J* = 2.5 Hz), 5.05 (2H, s), 4.24 (2H, s), 3.39 (2H, t, *J* = 6.0 Hz), 2.94 (2H, t, *J* = 6.0 Hz).

1.11.5. 2-(5-Chloro-2-nitropyridin-3-yl)-6-(4-fluorobenzyloxy)-1,2,3,4-tetrahydroisoquinoline (**12e**)

Yellow solid (yield 42%); ^{1}H NMR (500 MHz, $CDCl_3$) δ 7.91 (1H, d, *J* = 2.0 Hz), 7.52 (1H, d, *J* = 2.0 Hz), 7.40 (2H, dd, *J* = 5.5, 8.5 Hz), 7.08 (2H, t, *J* = 8.5 Hz), 7.02 (1H, d, *J* = 8.5 Hz), 6.83 (1H, dd, *J* = 2.5, 8.5 Hz), 6.78 (1H, d, *J* = 2.5 Hz), 5.02 (2H, s), 4.25 (2H, s), 3.40 (2H, t, *J* = 6.0 Hz), 2.96 (2H, t, *J* = 6.0 Hz).

1.11.6. 6-(4-Chlorobenzyloxy)-2-(5-chloro-2-nitropyridin-3-yl)-1,2,3,4-tetrahydroisoquinoline (**12f**)

Yellow solid (yield 39%); ^{1}H NMR (500 MHz, $CDCl_3$) δ 7.90 (1H, d, *J* = 2.0 Hz), 7.52 (1H, d, *J* = 2.0 Hz), 7.35 (4H, s), 7.02 (1H, d, *J* = 8.5 Hz), 6.82 (1H, dd, *J* = 2.5, 8.5 Hz), 6.77 (1H, d, *J* = 2.5 Hz), 5.02 (2H, s), 4.25 (2H, s), 3.39 (2H, t, *J* = 6.0 Hz), 2.95 (2H, t, *J* = 6.0 Hz).

1.11.7. 2-(5-Chloro-2-nitropyridin-3-yl)-6-(4-methoxybenzyloxy)-1,2,3,4-tetrahydroisoquinoline (**12g**)

Yellow solid (yield 39%); ^{1}H NMR (500 MHz, $CDCl_3$) δ 7.90 (1H, d, *J* = 2.0 Hz), 7.51 (1H, d, *J* = 2.0 Hz), 7.34 (2H, d, *J* = 8.5 Hz), 7.01 (1H, d, *J* = 8.0 Hz), 6.92 (2H, d, *J* = 8.5 Hz), 6.83 (1H, dd, *J* = 2.5, 8.5 Hz), 6.79 (1H, d, *J* = 2.5 Hz), 4.98 (2H, s), 4.24 (2H, s), 3.82 (3H, s), 3.39 (2H, t, *J* = 6.0 Hz), 2.95 (2H, t, *J* = 6.0 Hz).

*1.12. General Procedures for the Synthesis of Compounds **13a-d***

To a mixture of compound **12** (1.0 equiv.) and catechol (1.5 equiv.) in DMSO was added K_2CO_3 (2.0 equiv.) and 18-crown-6 (0.5 equiv.) under a nitrogen atmosphere. The mixture was heated at 90 ℃ for 3 h. After cooling to rt, the mixture was adjusted to pH 6 with NH_4Cl (aq) and extracted with EtOAc. The combined organic layers were dried over $MgSO_4$, filtered and concentrated in vacuo. The residue was purified by flash column chromatography (*n*-hexane/EtOAc) to provide compound **13**.

1.12.1. 5-(3,4-Dihydro-*1H*-isoquinolin-2-yl)-6-nitropyridin-3-yloxyphen-2-ol (**13a**)

Yellow solid (yield 49%); ^{1}H NMR (500 MHz, $CDCl_3$) δ 7.67 (1H, d, *J* = 2.5 Hz), 7.22-7.15 (4H, m), 7.09 (1H, dd, *J* = 1.5, 8.5 Hz), 7.05-7.04 (2H, m), 6.99 (1H, dd, *J* = 1.5, 8.0 Hz), 6.93 (1H, td, *J* = 1.5, 8.0 Hz), 5.72 (1H, s), 4.23 (2H, s), 3.38 (2H, t, *J* = 6.0 Hz), 2.96 (2H, t, *J* = 6.0 Hz).

1.12.2. 5-(6-Benzyloxy-3,4-dihydro-*1H*-isoquinolin-2-yl)-6-nitropyridin-3-yloxyphen-2-ol (**13d**)

Yellow solid (yield 39%); ^{1}H NMR (500 MHz, $CDCl_3$) δ 7.64 (1H, d, *J* = 2.0 Hz), 7.43-7.37 (4H, m), 7.32 (1H, t, *J* = 7.5 Hz), 7.16 (1H, td, *J* = 1.5, 7.5 Hz), 7.07 (1H, dd, *J* = 1.5, 7.5 Hz), 7.01 (1H, d, *J* = 2.5 Hz), 6.99-6.90 (3H, m), 6.80 (1H, dd, *J* = 2.5, 8.5 Hz), 6.77 (1H, d, *J* = 2.5 Hz), 5.81 (1H, br s), 5.04 (2H, s), 4.16 (2H, s), 3.33 (2H, t, *J* = 5.5 Hz), 2.90 (2H, t, *J* = 5.5 Hz).

*1.13. General Procedures for the Synthesis of Compounds **14***

To a mixture of compound **12** (1.0 equiv.) and resorcinol (1.5 equiv.) in DMSO was added K_2CO_3 (2.0 equiv.) and 18-crown-6 (0.5 equiv.) under a nitrogen atmosphere. The mixture was heated at 100 °C for 3 h. After cooling to rt, the mixture was adjusted to pH 6 with NH_4Cl (aq) and extracted with EtOAc. The combined organic layers were dried over $MgSO_4$, filtered and concentrated in vacuo. The residue was purified by flash column chromatography (*n*-hexane/EtOAc) to provide compound **14**.

1.13.1. 5-(3,4-Dihydro-*1H*-isoquinolin-2-yl)-6-nitropyridin-3-yloxyphen-3-ol (**14a**)

Yellow solid (yield 25%); ^{1}H NMR (500 MHz, $CDCl_3$) δ 7.67 (1H, d, *J* = 2.0 Hz), 7.26 (1H, t, *J* = 8.2 Hz), 7.20-7.15 (3H, m), 7.09 (1H, d, *J* = 2.0 Hz), 7.06 (1H, d, *J* = 6.6 Hz), 6.72 (1H, dd, *J* = 2.2, 8.2 Hz), 6.63 (1H, dd, *J* = 2.0, 8.2 Hz), 6.58 (1H, dd, *J* = 2.0, 2.2 Hz), 5.58 (1H, br s), 4.25 (2H, s), 3.39 (2H, t, *J* = 5.8 Hz), 2.97 (2H, t, *J* = 5.8 Hz).

1.13.2. 6-Nitro-5-(6-propoxy-3,4-dihydro-*1H*-isoquinolin-2-yl)pyridin-3-yloxyphen-3-ol (**14b**)

Yellow solid (yield 29%); ^{1}H NMR (500 MHz, $CDCl_3$) δ 7.67 (1H, d, *J* = 2.5 Hz), 7.26 (1H, t, *J* = 8.5 Hz), 7.06 (1H, d, *J* = 2.5 Hz), 6.96 (1H, d, *J* = 8.5 Hz), 6.75 (1H, dd, *J* = 2.5, 8.5 Hz), 6.72-6.69 (2H, m), 6.64 (1H, dd, *J* = 1.0, 8.0 Hz), 6.58 (1H, t, *J* = 2.5 Hz), 4.18 (2H, s), 3.90 (2H, t, *J* = 7.0 Hz), 3.36 (2H, t, *J* = 6.0 Hz), 2.93 (2H, t, *J* = 6.0 Hz), 1.80 (2H, sext., *J* = 7.0 Hz), 1.03 (3H, t, *J* = 7.0 Hz).

1.13.3. 5-(6-Cyclopropylmethoxy-3,4-dihydro-*1H*-isoquinolin-2-yl)-6-nitropyridin-3-yloxyphen-3-ol (**14c**)

Yellow solid (yield 28%); ^{1}H NMR (500 MHz, $CDCl_3$) δ 7.68 (1H, d, *J* = 2.0 Hz), 7.26 (1H, t, *J* = 8.0 Hz), 7.06 (1H, d, *J* = 2.0 Hz), 6.96 (1H, d, *J* = 8.5 Hz), 6.75 (1H, dd, *J* = 2.5, 8.0 Hz), 6.72-6.70 (2H, m), 6.64 (1H, d, *J* = 8.0 Hz), 6.58 (1H, t, *J* = 2.5 Hz), 4.18 (2H, s), 3.78 (2H, d, *J* = 7.0 Hz), 3.36 (2H, t, *J* = 5.5 Hz), 2.92 (2H, t, *J* = 5.5Hz), 1.30-1.22 (1H, m), 0.66-0.62 (2H, m), 0.34 (2H, dd, *J* = 4.5, 10.5 Hz).

1.13.4. 5-(6-Benzyloxy-3,4-dihydro-*1H*-isoquinolin-2-yl)-6-nitropyridin-3-yloxyphen-3-ol (**14d**)

Yellow solid (yield 74%); ^{1}H NMR (500 MHz, $CDCl_3$) δ 7.65 (1H, d, *J* = 2.5 Hz), 7.42-7.36 (4H, m), 7.31 (1H, t, *J* = 8.5 Hz), 7.23 (1H, t, *J* = 8.5 Hz), 7.05 (1H, d, *J* = 2.5 Hz), 6.95 (1H, d, *J* = 8.5 Hz), 6.80 (1H, dd, *J* = 2.5, 8.0 Hz), 6.77 (1H, d, *J* = 2.5 Hz), 6.72 (1H, dd, *J* = 2.5, 8.5Hz), 6.61-6.57 (2H, m), 5.04 (2H, s), 4.16 (2H, s), 3.34 (2H, t, *J* = 5.5 Hz), 2.91 (2H, t, *J* = 5.5Hz).

1.13.5. 5-[6-(4-Fluorobenzyloxy)-3,4-dihydro-*1H*-isoquinolin-2-yl]-6-nitropyridin-3-yloxyphen-3-ol (**14e**)

Yellow solid (yield 50%); ^{1}H NMR (500 MHz, $CDCl_3$) δ 7.67 (1H, d, *J* = 2.5 Hz), 7.39 (2H, dd, *J* = 5.5, 8.5 Hz), 7.23 (1H, t, *J* = 8.0 Hz), 7.08-7.05 (3H, m), 6.97 (1H, d, *J* = 8.5 Hz), 6.80 (1H, dd, *J* = 2.5, 8.0 Hz), 6.76 (1H, d, *J* = 2.5 Hz), 6.72 (1H, dd, *J* = 2.0, 8.5 Hz), 6.62 (1H, dd, *J* = 2.0, 8.0 Hz), 6.57 (1H, t, *J* = 2.0 Hz), 5.00 (2H, s), 4.18 (2H, s), 3.35 (2H, t, *J* = 6.0 Hz), 2.93 (2H, t, *J* = 6.0 Hz).

1.13.6. 5-[6-(4-Chlorobenzyloxy)-3,4-dihydro-*1H*-isoquinolin-2-yl]-6-nitropyridin-3-yloxyphen-3-ol (**14f**)

Yellow solid (yield 12%); ^{1}H NMR (500 MHz, $CDCl_3$) δ 7.69 (1H, d, *J* = 2.5 Hz), 7.36 (4H, s), 7.27 (1H, t, *J* = 8.5 Hz), 7.06 (1H, d, *J* = 2.5 Hz), 6.98 (1H, d, *J* = 8.0 Hz), 6.80 (1H, dd, *J* = 2.5, 8.5 Hz), 6.76 (1H, d, *J* = 2.0 Hz), 6.71 (1H, dd, *J* = 2.0, 8.0 Hz), 6.64 (1H, dd, *J* = 2.5, 8.5 Hz), 6.58 (1H, t, *J* = 2.5 Hz), 5.02 (2H, s), 4.19 (2H, s), 3.36 (2H, t, *J* = 6.0 Hz), 2.93 (2H, t, *J* = 6.0 Hz).

1.13.7. 5-[6-(4-Methoxybenzyloxy)-3,4-dihydro-*1H*-isoquinolin-2-yl]-6-nitropyridin-3-yloxyphen-3-ol (**14g**)

Yellow solid (yield 62%); ^{1}H NMR (500 MHz, $CDCl_3$) δ 7.68 (1H, d, *J* = 2.5 Hz), 7.35 (2H, d, *J* = 8.5 Hz), 7.25 (1H, t, *J* = 8.5 Hz), 7.05 (1H, d, *J* = 2.5 Hz), 6.97 (1H, d, *J* = 8.5 Hz), 6.92 (2H, d, *J* = 8.5 Hz), 6.81 (1H, dd, *J* = 2.0, 8.0 Hz), 6.77 (1H, d, *J* = 2.0 Hz), 6.72 (1H, dd, *J* = 2.5, 8.0 Hz), 6.62 (1H, dd, *J* = 2.5, 8.0 Hz), 6.58 (1H, t, *J* = 2.0 Hz), 4.97 (2H, s), 4.18 (2H, s), 3.82 (3H, s), 3.36 (2H, t, *J* = 5.5 Hz), 2.93 (2H, t, *J* = 5.5 Hz).

*1.14. General Procedures for the Synthesis of Compounds **15** and **16***

To a mixture of compound **13** or **14** (1.0 equiv.) in DCM was added TEA (2.0 equiv.) under a nitrogen atmosphere. The mixture was stirred at rt for 10 min, and then acryloyl chloride (2.0 equiv.) was added. After stirring for 1 h, the reaction mixture was neutralized with $NaHCO_3$ (aq) and extracted with DCM. The combined organic layers were dried over $MgSO_4$, filtered and concentrated in vacuo. The residue was purified by flash column chromatography (*n*-hexane/EtOAc) to provide **15** or **16**.

1.14.1. Acrylic Acid 2-[5-(3,4-dihydro-*1H*-isoquinolin-2-yl)-6-nitropyridin-3-yloxy]phenyl Ester (**15a**)

Yellow solid (yield 77%); ^{1}H NMR (500 MHz, $CDCl_3$) δ 7.71 (1H, d, *J* = 2.5 Hz), 7.36-7.30 (3H, m), 7.21-7.13 (4H, m), 7.05 (1H, d, *J* = 6.5 Hz), 7.01 (1H, d, *J* = 2.5 Hz), 6.47 (1H, dd, *J* = 1.0, 17.0 Hz), 6.18 (1H, dd, *J* = 10.5, 17.0 Hz), 5.95 (1H, dd, *J* = 1.0, 10.5 Hz), 4.21 (2H, s), 3.37 (2H, t, *J* = 5.5 Hz), 2.96 (2H, t, *J* = 5.5 Hz).

1.14.2. Acrylic Acid 2-[5-(6-benzyloxy-3,4-dihydro-*1H*-isoquinolin-2-yl)-6-nitropyridin-3-yloxy]phenyl Ester (**15d**)

Yellow solid (yield 30%); ^{1}H NMR (500 MHz, $CDCl_3$) δ 7.70 (1H, d, *J* = 2.0 Hz), 7.42-9.36 (4H, m), 7.33-7.25 (4H, m), 7.13 (1H, dd, *J* = 3.0, 6.0 Hz), 6.99 (1H, d, *J* = 2.5 Hz), 6.95 (1H, d, *J* = 8.5 Hz), 6.80 (1H, dd, *J* = 2.5, 8.5 Hz), 6.77 (1H, d, *J* = 2.5 Hz), 6.46 (1H, dd, *J* = 1.0, 17.0 Hz), 6.18 (1H, dd, *J* = 10.5, 17.0 Hz), 5.94 (1H, dd, *J* = 1.0, 10.5 Hz), 5.04 (2H, s), 4.14 (2H, s), 3.33 (2H, t, *J* = 5.5 Hz), 2.91 (2H, t, *J* = 5.5 Hz).

1.14.3. Acrylic Acid 3-[5-(3,4-dihydro-*1H*-isoquinolin-2-yl)-6-nitropyridin-3-yloxy]phenyl Ester (**16a**)

Yellow solid (yield 99%); ^{1}H NMR (500 MHz, $CDCl_3$) δ 7.75 (1H, d, *J* = 2.5 Hz), 7.45 (1H, t, *J* = 8.0 Hz), 7.21-7.16 (3H, m), 7.12 (1H, d, *J* = 2.0 Hz), 7.09 (1H, d, *J* = 6.5 Hz), 7.04 (1H, dd, *J* = 1.5, 8.0 Hz), 6.99 (1H, dd, *J* = 1.5, 8.0 Hz), 6.93 (1H, t, *J* = 1.5 Hz), 6.62 (1H, d, *J* = 17.5 Hz), 6.32 (1H, dd, *J* = 10.5, 17.5 Hz), 6.05 (1H, d, *J* = 10.5 Hz), 4.26 (2H, s), 3.40 (2H, t, *J* = 6.0 Hz), 2.98 (2H, t, *J* = 6.0 Hz).

1.14.4. Acrylic Acid 3-[6-nitro-5-(6-propoxy-3,4-dihydro-*1H*-isoquinolin-2-yl)pyridin-3-yloxy]phenyl Ester (**16b**)

Yellow solid (yield 62%); ^{1}H NMR (500 MHz, $CDCl_3$) δ 7.73 (1H, d, *J* = 2.5 Hz), 7.44 (1H, t, *J* = 8.5 Hz), 7.10 (1H, d, *J* = 2.5 Hz), 7.03 (1H, ddd, *J* = 1.0, 2.5, 8.5 Hz), 6.99-6.97 (2H, m), 6.92 (1H, t, *J* = 2.5 Hz), 6.74 (1H, dd, *J* = 2.5, 8.5 Hz), 6.69 (1H, d, *J* = 2.5 Hz), 6.61 (1H, dd, *J* = 1.5, 17.5 Hz), 6.31 (1H, dd, *J* = 10.5, 17.5 Hz), 6.05 (1H, dd, *J* = 1.5, 10.5 Hz), 4.20 (2H, s), 3.90 (2H, t, *J* = 7.0 Hz), 3.37 (2H, t, *J* = 6.0 Hz), 2.93 (2H, t, *J* = 6.0 Hz), 1.80 (2H, sext., *J* = 7.0 Hz), 1.03 (3H, t, *J* = 7.0 Hz).

1.14.5. Acrylic Acid 3-[5-(6-cyclopropylmethoxy-3,4-dihydro-*1H*-isoquinolin-2-yl)-6-nitropyridin-3-yloxy]phenyl Ester (**16c**)

Yellow solid (yield 82%); ^{1}H NMR (500 MHz, $CDCl_3$) δ 7.73 (1H, d, *J* = 2.5 Hz), 7.44 (1H, t, *J* = 8.0 Hz), 7.10 (1H, d, *J* = 2.5 Hz), 7.03 (1H, dd, *J* = 2.0, 8.0 Hz), 6.98 (2H, d, *J* = 9.5 Hz), 6.92 (1H, t, *J* = 2.5 Hz), 6.75 (1H, dd, *J* = 2.5, 8.5 Hz), 6.69 (1H, d, *J* = 2.5 Hz), 6.61 (1H, dd, *J* = 1.0, 17.5 Hz), 6.31 (1H, dd, *J* = 10.5, 17.0 Hz), 6.05 (1H, dd, *J* = 1.0, 10.5 Hz), 4.20 (2H, s), 3.78 (2H, d, *J* = 6.5 Hz), 3.36 (2H, t, *J* = 6.0 Hz), 2.93 (2H, t, *J* = 6.0 Hz), 1.29-1.23 (1H, m), 0.66-0.62 (2H, m), 0.34 (2H, dd, *J* = 4.5, 10.5 Hz).

1.14.6. Acrylic Acid 3-[5-(6-Benzyloxy-3,4-dihydro-*1H*-isoquinolin-2-yl)-6-nitropyridin-3-yloxy]phenyl Ester (**16d**)

Yellow solid (yield 34%); ^{1}H NMR (500 MHz, $CDCl_3$) δ 7.73 (1H, d, *J* = 2.5 Hz), 7.45-7.36 (5H, m), 7.32 (1H, t, *J* = 7.5 Hz), 7.10 (1H, d, *J* = 2.0 Hz), 7.03 (1H, ddd, *J* = 0.5, 2.0, 8.0 Hz), 7.00-6.97 (2H, m), 6.92 (1H, t, *J* = 2.5 Hz), 6.82 (1H, dd, *J* = 2.5, 8.5 Hz), 6.78 (1H, d, *J* = 2.5 Hz), 6.61 (1H, dd, *J* = 1.0, 17.5 Hz), 6.31 (1H, dd, *J* = 10.5, 17.5 Hz), 6.04 (1H, dd, *J* = 1.0, 10.5 Hz), 5.05 (2H, s), 4.20 (2H, s), 3.36 (2H, t, *J* = 5.5 Hz), 2.93 (2H, t, *J* = 5.5 Hz).

1.14.7. Acrylic Acid 3-{5-[6-(4-fluorobenzyloxy)-3,4-dihydro-*1H*-isoquinolin-2-yl]-6-nitropyridin-3-yloxy}phenyl Ester (**16e**)

Yellow solid (yield 84%); ^{1}H NMR (500 MHz, $CDCl_3$) δ 7.74 (1H, d, *J* = 2.0 Hz), 7.44 (1H, t, *J* = 8.0 Hz), 7.39 (2H, dd, *J* = 5.5, 8.5 Hz), 7.10 (1H, d, *J* = 2.0 Hz), 7.07 (2H, t, *J* = 8.5 Hz), 7.04-6.98 (3H, m), 6.80 (1H, dd, *J* = 2.5, 8.5 Hz), 6.76 (1H, d, *J* = 2.5 Hz), 6.61 (1H, dd, *J* = 1.0, 17.5 Hz), 6.31 (1H, dd, *J* = 10.5, 17.5 Hz), 6.05 (1H, dd, *J* = 1.0, 10.5 Hz), 5.00 (2H, s), 4.20 (2H, s), 3.37 (2H, t, *J* = 5.5 Hz), 2.94 (2H, t, *J* = 5.5 Hz).

1.14.8. Acrylic Acid 3-{5-[6-(4-chlorobenzyloxy)-3,4-dihydro-*1H*-isoquinolin-2-yl]-6-nitropyridin-3-yloxy}phenyl Ester (**16f**)

Yellow solid (yield 84%); ^{1}H NMR (500 MHz, $CDCl_3$) δ 7.74 (1H, d, *J* = 2.0 Hz), 7.44 (1H, t, *J* = 8.5 Hz), 7.36 (4H, s), 7.10 (1H, d, *J* = 2.0 Hz), 7.04-6.99 (3H, m), 6.92 (1H, d, *J* = 2.5 Hz), 6.80 (1H, dd, *J* = 2.5, 8.5 Hz), 6.76 (1H, s), 6.61 (1H, d, *J* = 17.5 Hz), 6.31 (1H, dd, *J* = 10.5, 17.5 Hz), 6.05 (1H, d, *J* = 10.5 Hz), 5.02 (2H, s), 4.20 (2H, s), 3.37 (2H, t, *J* = 5.5 Hz), 2.94 (2H, t, *J* = 5.5Hz).

1.14.9. Acrylic Acid 3-{5-[6-(4-methoxybenzyloxy)-3,4-dihydro-*1H*-isoquinolin-2-yl]-6-nitropyridin-3-yloxy}phenyl Ester (**16g**)

Yellow solid (yield 90%); ^{1}H NMR (500 MHz, $CDCl_3$) δ 7.72 (1H, d, *J* = 2.0 Hz), 7.43 (1H, t, *J* = 8.0 Hz), 7.34 (2H, d, *J* = 8.5 Hz), 7.10 (1H, d, *J* = 2.0 Hz), 7.03 (1H, dd, *J* = 2.0, 8.5 Hz), 6.98 (2H, d, *J* = 8.5 Hz), 6.93-6.90 (3H, m), 6.80 (1H, dd, *J* = 2.5, 8.5 Hz), 6.76 (1H, d, *J* = 2.0 Hz), 6.60 (1H, d, *J* = 17.0 Hz), 6.30 (1H, dd, *J* = 10.5, 17.0 Hz), 6.04 (1H, d, *J* = 10.5 Hz), 4.96 (2H, s), 4.19 (2H, s), 3.80 (3H, s), 3.35 (2H, t, *J* = 5.5 Hz), 2.92 (2H, t, *J* = 5.5 Hz).

2. A List of BTK Crystal Structures from Protein Data Bank (Accessed on 10th August 2020)

3GEN, 3K54, 3PIX, 3PIY, 3PIZ, 3PJ1, 3PJ2, 3PJ3, 3T9T, 4NWM, 4OT5, 4OT6, 4OTQ, 4OTR, 3OCS, 4RFY, 4RFZ, 4RG0, 4YHF, 4Y95, 4Z3V, 4ZLY, 4ZLZ, 5BPY, 5BQ0, 4OTF, 4RX5, 5FBN, 5FBO, 5KUP,

5JRS, 5T18, 5VGO, 5P9F, 5P9M, 5P9K, 5P9L, 5P9H, 5P9I, 5P9J, 5P9G, 5J87, 5VFI, 5ZZ4, 6DI3, 6DI5, 6DI9, 6DI0, 6DI1, 6AUA, 6AUB, 6E4F, 6BIK, 6EP9, 6BLN, 6BKW, 6BKH, 6BKE, 6O8I, 6OMU, 6J6M, 6HRP, 6HRT, 6MNY, 6S90, 6N9P, 6NFH, 6NFI, 6NZM, 6TFP, 6XE4, 6X3O, 6X3P, 6X3N.
